# Functional cyclic disulfides enable multi-pathway degradable polyvinyls

**DOI:** 10.1039/d6sc04278c

**Published:** 2026-08-25

**Authors:** Alessandro Magro, Elena Avanzini, Daniele Rizzi, Giovanni Gobbo, Francesco M. Ara, Rachele Foladore, Alessandro Bertolo, Cristian Pezzato, Gianluca Gazzola, Francesca Lorandi, Edmondo M. Benetti

**Affiliations:** a Laboratory for Macromolecular and Organic Chemistry, Department of Chemical Sciences, University of Padova Via Marzolo 1 35131 Padova Italy edmondo.benetti@unipd.it francesca.lorandi@unipd.it +390498275135 +390498275232; b Amethyst Lab SRL Via Tiberio Deciani 2 33100 Udine Italy

## Abstract

Copolymerization of vinyl monomers with functional seven-membered cyclic disulfides enables the synthesis of polyvinyl esters capable of undergoing distinct degradation pathways. More generally, the incorporation of cleavable linkages into polyvinyl backbones *via* radical copolymerization provides a powerful strategy toward degradable materials while preserving the versatility and scalability of vinyl polymerization. Here, we introduce molecularly designed seven-membered cyclic disulfides as comonomers for radical copolymerization with vinyl esters, enabling incorporation of both disulfide and ester functionalities along polyvinyl backbones. In particular, the ester-functionalized monomer 1,4,5-oxadithiepan-2-one (ODP) affords copolymers that undergo reductive disulfide cleavage, glutathione (GSH)-mediated thiol-disulfide exchange, and hydrolytic backbone fragmentation under mildly basic conditions. The copolymerization behavior of seven-membered cyclic disulfides with vinyl esters provides insight into structure-reactivity relationships in radical ring-opening copolymerization, while remaining compatible with photoiniferter RAFT/MADIX polymerization. Water-soluble copolymers derived from diethylene glycol vinyl esters form highly hydrated, protein-repellent films, combining bioinert interfacial properties with susceptibility to degradation through orthogonal chemical pathways. These results establish functional seven-membered cyclic disulfides as a versatile platform for the synthesis of degradable and potentially biodegradable polyvinyl materials.

## Abstract 2

Functional seven-membered cyclic disulfides enable the radical copolymerization of vinyl esters into polyvinyls degradable through multiple pathways.

## Graphical abstract

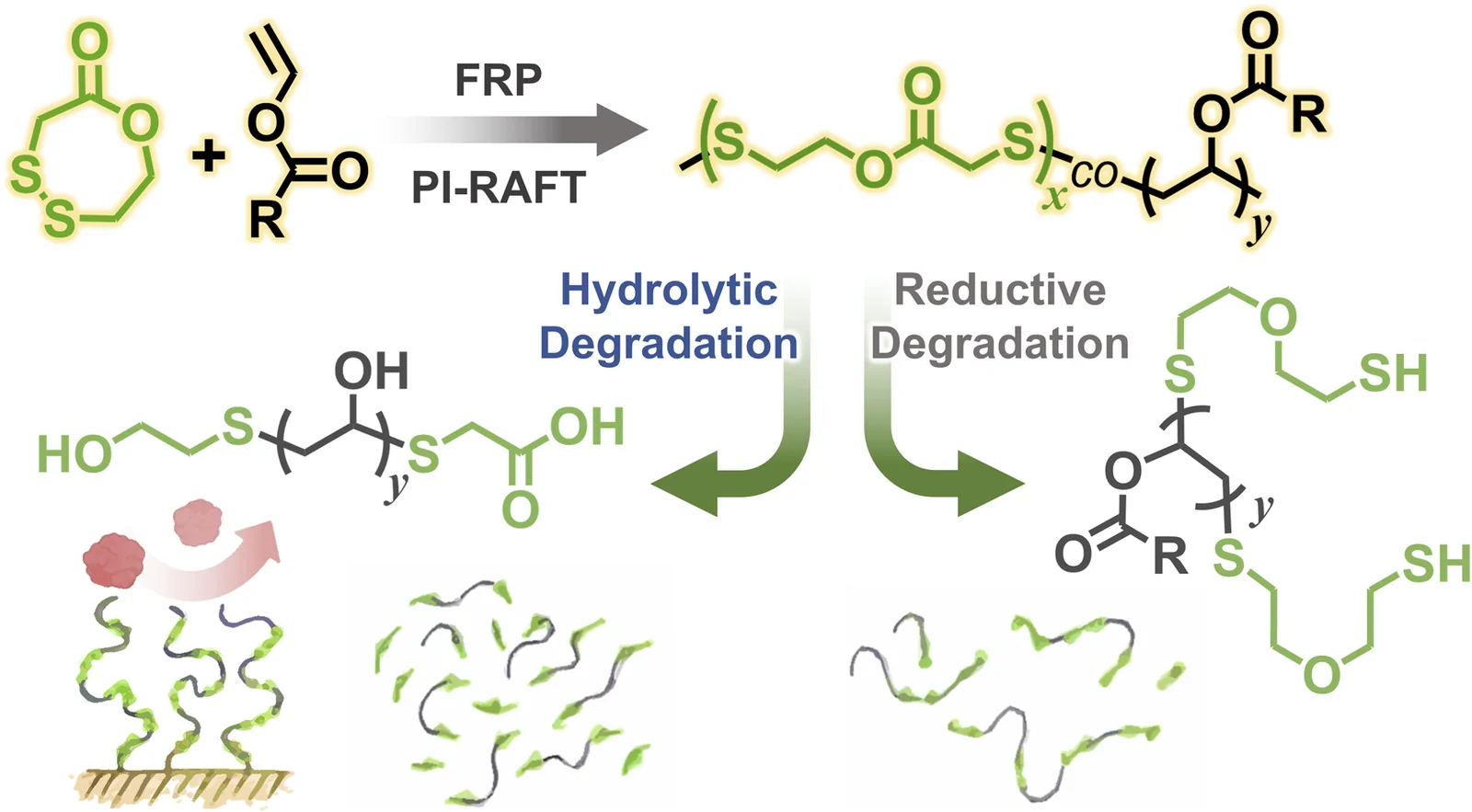

## Introduction

Embedding cleavable linkages directly into polyvinyl backbones *via* radical copolymerization is a particularly attractive strategy to generate commodity plastics that are both degradable/recyclable and that could be virtually generated through scalable processes that are industrially relevant.^1–4^ Among the various labile bonds that can be incorporated along polyvinyl chains, disulfides offer unique advantages, combining redox-responsiveness,^5–7^ dynamic exchange,^7–9^ and mild cleavage conditions.^10–12^

Radical copolymerization of vinyl monomers with cyclic disulfides, such as five-membered 1,2-dithiolanes and six-membered 1,2-dithianes, has become especially appealing and versatile to generate disulfide-containing polyvinyls, since the strain relief during ring-opening makes cyclic disulfides reactive and prone to copolymerization toward diverse monomers.^13–15^ Pioneering radical copolymerization of lipoamide—a bioderived 1,2-dithiolane-based co-factor—with styrene (St), acrylates, vinyl acetate (VAc) and acrylonitrile (AN) already demonstrated that vinyl radicals can trigger disulfide's radical ring-opening to yield backbone sulfide–disulfide motifs.^16^ Later on, Tsarevsky *et al.* established that α-lipoic acid (LA)—the parent metabolite of lipoamide—and its alkyl esters undergo radical ring-opening during free-radical copolymerization with acrylate monomers, leading to copolymers containing substantial fractions of disulfide linkages along their main chain.^17^ These copolymers degrade rapidly under reductive conditions, validating cyclic disulfides as efficient comonomers for programmable backbone cleavability.^17,18^ Thanks to the more recent, fundamental works by the groups of Hawker and Bates, LA has progressively emerged as a privileged cyclic disulfide monomer owing to its commercial availability, bio-based origin, and benign toxicological profile.^18–20^ The advent of controlled radical polymerizations (CRP) has expanded its application as co-monomer, giving access to statistical copolymers with fine control over composition, molar mass, and architecture, in which LA-derived disulfides are precisely distributed along an array of polyvinyl backbones, also including polystyrenes, water soluble polyacrylates and polyacrylamides.^18,21,22^

More recent research enlarged the choice of cyclic disulfide comonomers to the less strained, 6-membered ring 1,2-dithianes.^15^ These systems can be successfully copolymerized with less-activated monomers (LAMs), such as VAc, enabling not only chain degradation *via* disulfide reduction but also acid-catalyzed hydrolytic cleavage of hemithioacetal linkages. The latter are formed along the polymer backbone when vinyl ester radicals cross-propagate with cyclic disulfide species.

Despite the versatility and broad monomer scope enabled by the application of 1,2-dithiolanes and dithianes, polymers generated through their copolymerization with vinyl monomers could not introduce classically hydrolysable ester functionalities, which are susceptible to hydrolysis under basic/physiological conditions and are expected to promote degradation under environmentally relevant settings (*i.e.*, through esterases or lipases). More generally, these approaches are limited to introducing thioether (C–S–C) and disulfide (S–S) linkages along the carbon–carbon polyvinyl backbone, restricting access to functional groups that could enable alternative degradation pathways.

Motivated by these limitations, we concentrated on exploring alternative co-monomers that retain the characteristic reactivity of cyclic disulfides toward vinyl radicals while incorporating additional heteroatoms or functional moieties. Such structures would enable the introduction of new functionalities along the polyvinyl backbone, including groups capable of undergoing hydrolysis in a manner analogous to (bio)degradable polyesters prepared *via* ionic or coordinative ring-opening polymerization of lactones.

A source of inspiration came from fundamental works dating back to the early 50's, when Tobolsky *et al.* first demonstrated that 1,4,5-oxadithiacycloheptane (ODC)—a 7-membered ring disulfide featuring an endocyclic ether linkage—can generate diradical species reactive toward (meth)acrylates and styrenes.^23,24^ Stockmayer *et al.*^25^ simultaneously proved that ODC could copolymerize through radical ring opening with VAc, thereby establishing a conceptual foundation for expanding the chemical toolbox of degradable polyvinyl materials through the use of heterofunctional cyclic disulfides.

**Fig. 1:**
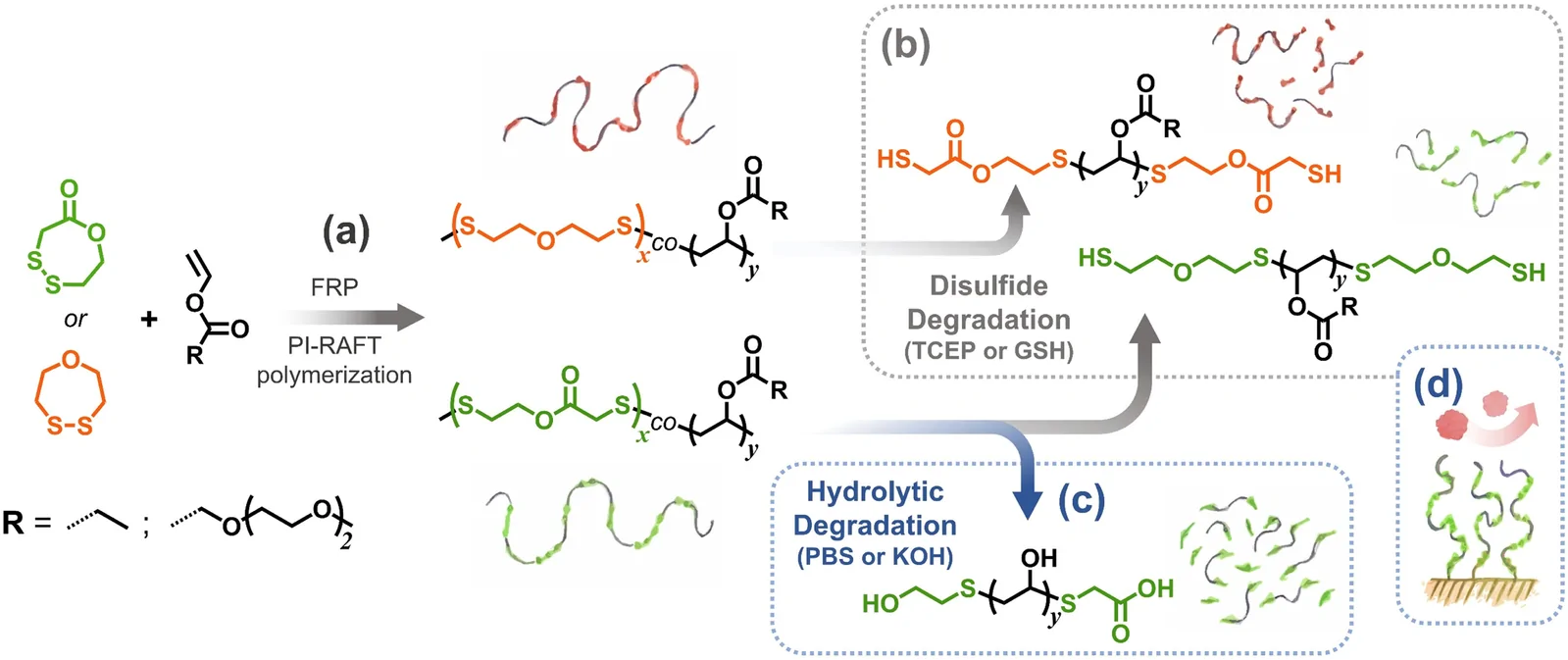
General strategy toward degradable polyvinyls by exploiting the radical copolymerization of functional cyclic disulfide monomers. (a) Copolymerization of ODC (orange) and ODP (green) with vinyl esters through free radical polymerization (FRP) and photoiniferter RAFT (PI-RAFT) polymerization conditions; (b) disulfide degradation of the resulting copolymers using a mild reductant or by exploiting thiol-disulfide exchange with GSH; (c) hydrolytic degradation under basic conditions or phosphate buffer saline (PBS) with pH = 7.4; (d) bioinert behavior of water-soluble and degradable vinyl copolymers.

The curiosity sparked by these pioneering studies, together with the renewed interest in cyclic disulfides for (controlled) radical polymerization, prompted us to investigate new synthetic routes aimed at producing polyvinyl copolymers that are not only redox-responsive and degradable but also hydrolysable under environmentally relevant conditions. In this context, we examined the radical copolymerization behavior of both ODC and 1,4,5-oxadithiepan-2-one (ODP) (Fig. 1) with a series of representative vinyl esters. Notably, ODP is a seven-membered cyclic disulfide structurally related to ODC but distinguished by the incorporation of an ester functionality within the ring, enabling an additional hydrolytic degradation pathway alongside redox-triggered disulfide cleavage.

Vinyl ester co-monomers included VAc and (diethylene glycol)vinyl ester (DEGVE), which provides water soluble and bioinert polyvinyls with properties that match those displayed by the most widespread poly(oligoethylene glycol)acrylates and methacrylates (POEGA and POEGMA, respectively).^26,27^ The resulting copolymers could be successfully degraded through multiple pathways, including cleavage of disulfide linkages *via* reducing agents and glutathione (GSH)-mediated thiol-disulfide exchange, as well as hydrolysis of ester functions within basic media and physiological buffer solutions.

## Results and discussion

ODC and ODP were synthesized *via* intramolecular oxidation of the corresponding terminal dithiols, following previously reported procedures.^28,29^ Both monomers were obtained as pale-yellow oils in 60–70% yields (Fig. S1–S5).

We subsequently investigated the ability of these monomers to undergo radical ring opening polymerization (rROP) to yield homopolymers in the presence of a radical initiator (Table S1). The rROP of ODC was performed both in bulk and in toluene ([ODC] = 3 M) at 80 °C using 2-2′-azobisisobutyronitrile (AIBN) as initiator (2 mol% relative to the monomer). After 24 h, ODC conversions of 62% and 89% were determined by ^1^H NMR for the polymerization in bulk and in toluene, respectively (Table S1). Size exclusion chromatography (SEC) of purified polymers showed molar masses of *M*_n_ = 31.0 and 35.7 kg mol^−1^ (Fig. S6). The rROP of ODP was conducted in bulk at 80 °C, in the presence of 2 mol% AIBN. The monomer reached 92% conversion after 8 h of reaction, giving a polymer with *M*_n_ = 21.2 kg mol^−1^ upon purification (Table S1 and Fig. S7).

**Fig. 2:**
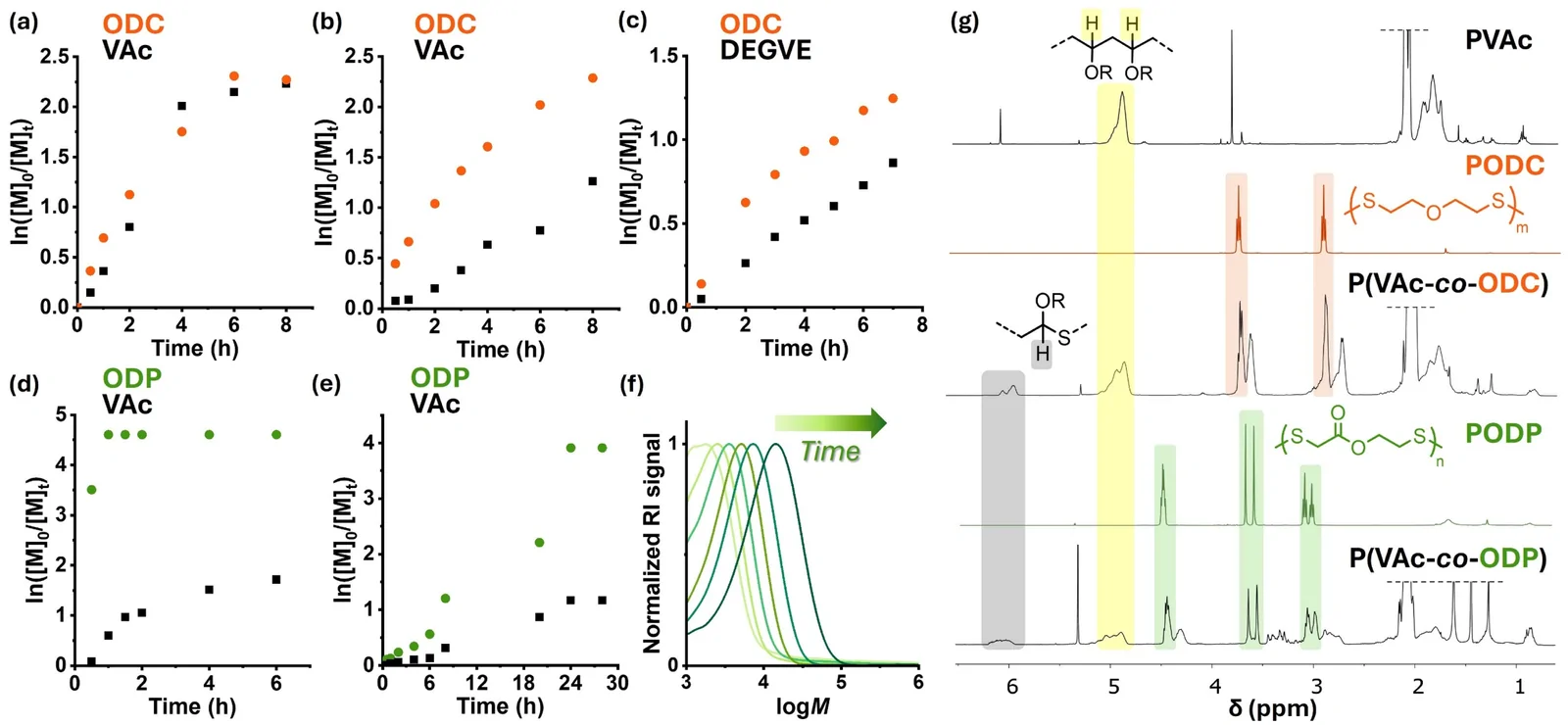
Copolymerization of ODC or ODP with vinyl monomers. (a and b) Copolymerization of ODC and VAc *via* FRP with initial comonomer feed of [VAc]_0_ : [ODC]_0_ = (a) 95 : 5, and (b) 85 : 15. (c) Copolymerization of ODC with di(ethylene glycol) vinyl ester (DEGVE) with comonomer feed [DEGVE]_0_ : [ODC]_0_ = 85 : 15. (d and e) Copolymerization of ODP and VAc with comonomer feed [VAc]_0_ : [ODP]_0_ = 85 : 15 *via* (d) FRP, and (e) photoiniferter RAFT/MADIX process. (f) SEC traces showing the evolution of copolymer molar mass during copolymerization of ODP and VAc *via* photoiniferter RAFT/MADIX process. (g) ^1^H NMR spectra in CDCl_3_ of PVAc, PODC, PODP, and of the copolymers P(VAc-*co*-ODC) and P(VAc-*co*-ODP) highlighting the signals corresponding to homo-oligomeric/polymeric fragments and the signal at *δ* ∼ 6 ppm demonstrating the presence of ODC–VAc and ODP–VAc diads.

Having demonstrated that heterofunctional seven-membered cyclic disulfides are reactive toward rROP, we later on investigated their copolymerization with vinyl esters, initially concentrating on copolymerizing ODC with VAc. This copolymerization was conducted in bulk at 70 °C with an initial feed ratio of [VAc]_0_ : [ODC]_0_ = 95 : 5. Relevantly, ODC and VAc reacted at comparable rates reaching ∼90% conversion after 7–8 h (Table 1 and Fig. 2a) and providing copolymers with *M*_n_ = 30.3 kg mol^−1^, as measured by SEC (Fig. S8). When the relative content of ODC in the feed was increased to 15 mol% this was consumed at a slightly higher rate compared to VAc (Fig. 2b), whereas both monomers reached conversion >70% after 8 h (Table 1), yielding copolymers with *M*_n_ = 22.2 kg mol^−1^. The successful incorporation of ODC units within the polyvinyl backbone was confirmed by ^1^H NMR analysis of the obtained copolymers (Fig. 2g, Fig. S9 and S10). The ^1^H NMR spectrum of PODC homopolymer showed two triplets at *δ* = 3.85 and 2.95 ppm, which were ascribed to the methylene protons from the repeating unit of the oligo/polydisulfide chains (–O–C**H**_**2**_–C**H**_**2**_–S–S–C**H**_**2**_–C**H**_**2**_–O–). In the ^1^H NMR spectra of P(VAc-*co*-ODC) copolymers, these signals persisted, as originating from ODC–ODC diads (Fig. 2g), while co-existing with two additional signals—shifted downfield to *δ* = 3.65 and 2.75 ppm—correlated with ODC–VAc diads (–O–C**H**_**2**_–C**H**_**2**_–S–CH_2_–CH(OOCCH_3_)–) and generated from cross-propagation.^28^ In addition, the presence of VAc–ODC diads from cross-propagation by vinyl acetate (macro)radicals was also confirmed by the appearance of the signal at *δ* = 6 ppm, which was attributed to the methine proton of a VAc unit linked to a S atom (–CH_2_–C**H**(OOCCH_3_)–S–CH_2_–CH_2_–O–, Fig. 2g).^30^^1^H NMR characterization also enabled to estimate the incorporated fractions of ODC (*F*_ODC_) within the copolymers, which were 13 and 32%—significantly higher values compared to the corresponding [ODC]_0_ in the feeds (5 and 15 mol%, respectively). This enrichment reflected the higher reactivity of ODC relative to VAc during copolymerization. Confirmation of the incorporation of ODC units into the PVAc backbone was additionally provided by DOSY NMR analysis, which revealed a single diffusion coefficient for P(VAc-*co*-ODC) copolymer species (Fig. S11).

**Table 1:** Copolymerization of cyclic disulfide (*M*_S–S_) and vinyl ester (*M*_vin_) monomers^a^

Entry	*M*_S–S_	*M*_vin_	[*M*_s–s_]_0_ : [*M*_vin_]_0_	*t* (h)	Conv. *M*_S–S_^b^ (%)	Conv. *M*_vin_^b^ (%)	*F*_ODC or ODP_ c (mol%)	*M*_n,th_ d (kg mol^−1^)	*M*_n_ e (kg mol^−1^)	*Đ* e
1^f^	ODC	VAc	5 : 95	8	90	89	13	—	30.3	1.94
2^f^		VAc	15 : 85	8	90	72	32	—	22.2	2.23
3		DEGVE	15 : 85	7	71	58	19	—	12.9	1.81
4^g^		VAc	15 : 85	30	94	79	34	11.8	10.3	1.84
5^g^		DEGVE	15 : 85	24	77	62	24	18.7	12.4	1.70
6	ODP	VAc	5 : 95	6	>99	90	8	—	28.6	2.55
7		VAc	15 : 85	6	>99	90	22	—	14.3	3.79
8^g^		VAc	5 : 95	28	>99	89	4	13.5	14.1	1.46
9^g^		VAc	15 : 85	28	98	69	32	11.0	9.9	1.75
10^g^		DEGVE	15 : 85	22	70	25	18	9.0	4.5	1.85

a Copolymerizations were performed in bulk at *T* = 70 °C with 2 mol% AIBN relative to the total monomer concentration.

b Determined by ^1^H NMR.

c Determined by ^1^H NMR on the purified copolymers.

d Calculated as *M*_n,th_ = [(Conv. *M*_S–S_) × DP_T,S–S_ × MM_S–S_] + [(Conv. *M*_vin_) × DP_T,vin_ × MM_vin_] + MM_CTA_.

e Measured by SEC using THF as eluent for *M*_vin_ = VAc, or DMF (with 1 g L^−1^ LiBr) as eluent for *M*_vin_ = DEGVE.

f 0.5 mol% AIBN relative to the total monomer concentration.

g Copolymerizations were performed in bulk at *T* ∼ 30 °C, under blue light irradiation: *λ*_max_ = 427 nm, with ([*M*_S–S_] + [*M*_vin_]) : [CTA] = 150 : 1.

We also explored the copolymerization of ODC with di(ethylene glycol) vinyl ester (DEGVE) (Fig. S12), providing access to water soluble and bioinert poly(vinyl ester)s—alternative materials to the more widely studied oligo(ethylene glycol) (OEG)-bearing poly(meth)acrylates.^26,27^ A comonomer feed ratio of [DEGVE]_0_ : [ODC]_0_ = 85 : 15, subjected to bulk copolymerization at 70 °C, successfully yielded P(DEGVE-*co*-ODC) with *M*_n_ = 12.9 kg mol^−1^ and featuring *F*_ODC_ = 19 mol% (Table 1, Fig. 2c, S13 and S14).

The synthesis of poly(vinyl ester)-based copolymers including ODC could also be accomplished by exploiting RAFT/MADIX process, using *O*-ethyl-*S*-(1-methoxycarbony)ethyldithiocarbonate (X1) as photoiniferter (Fig. S15) and blue light irradiation (*λ*_max_ = 427 nm, 70 mW cm^−2^). As it was expected from a controlled radical polymerization process, during RAFT/MADIX photoiniferter copolymerization of VAc/ODC ([VAc]_0_ : [ODC]_0_ = 85 : 15) the molar mass progressively increased with the irradiation time (Fig. S16 and S17), whereas both VAc and ODC were converted, respectively reaching 79 and 94% conversion after 30 h of reaction (Table 1 and Fig. S18). The resulting P(VAc-*co*-ODC) showed a *M*_n_ = 10.3 kg mol^−1^ (*Đ* = 1.84) with *F*_ODC_ = 34 mol% (Fig. S19). RAFT/MADIX photoiniferter process was also effective for the synthesis of P(DEGVE-*co*-ODC), which could be obtained from monomer feed ratio of [DEGVE]_0_ : [ODC]_0_ = 85 : 15, following 24 h of blue light irradiation, reaching *M*_n_ = 12.4 kg mol^−1^ (*Đ* = 1.70) with *F*_ODC_ = 24 mol% (Table 1 and Fig. S20).

Subsequently, we investigated the copolymerization of vinyl esters with ODP—the seven-membered cyclic disulfide designed to introduce ester functionalities along the polyvinyl backbones. The free radical copolymerization of VAc and ODP was performed using comonomer feed ratios of [VAc]_0_ : [ODP]_0_ = 95 : 5 and 85 : 15 (Table 1). In both cases, ODP was consumed faster than VAc. Quantitative conversion of ODP was reached within 2 h, while the conversion of VAc reached ∼90% after 5–6 h (Fig. 2d and S21). The different polymerization rates of the two comonomers indicate that ODP is more reactive than VAc, leading to marked compositional drifts within polymer chains. Notably, ODP appears to be more reactive than ODC, which was consumed just at a slightly higher rate than VAc under similar copolymerization conditions. SEC analysis of the purified products showed monomodal traces with *Đ* > 2 (Fig. S22). Despite their different reactivity, the incorporation of ODP units within polyvinyl backbones was clearly evidenced by ^1^H NMR (Fig. 2g and S23–S25), where various signals attributable to protons of adjacent VAc and ODP units were observed, in analogy to what was previously discussed for ODC. It should be noted that the asymmetric structure of ODP leads to numerous signals for each set of –C**H** and –C**H**_**2**_ protons in both comonomers due to “head-to-tail” and “head-to-head” additions of ODP units,^29^ which further complicate the sequence-dependent pattern. The P(VAc-*co*-ODP) copolymers exhibited *F*_ODP_ = 8 and 22 mol%.

Interestingly, ^1^H NMR spectra collected during the polymerization revealed that the cross-propagation signal at *δ* ∼ 6 ppm, –CH_2_–C**H**(OOCCH_3_)–S–, increased over time even after ODP was entirely consumed (Fig. S23). This observation suggests that during the homopropagation of the remaining VAc units chain transfer reactions occurred at the disulfide bonds of ODP-rich chains. In order to confirm this phenomenon, we performed a FRP of VAc in the presence of PODP. A small signal at *δ* ∼ 6 ppm was already detectable after 30 minutes of polymerization and it increased in intensity as the reaction progressed (Fig. S26), clearly indicating that S–S bonds acted as chain transfer sites, consistent with a reported chain transfer constant *C*_T_ = 0.89 at 60 °C for the S–S group of thiocol H(SCH_2_CH_2_OCH_2_OCH_2_CH_2_S)_*n*_H in the FRP of VAc.^30^

Having established the higher reactivity of ODP compared to VAc in FRP at *T* = 70 °C, we then turned to a photoiniferter RAFT/MADIX process to investigate whether the lower reaction temperature could improve the reactivity match. Copolymerization kinetics for initial comonomer feed ratios of [VAc]_0_ : [ODP]_0_ = 95 : 5 and 85 : 15 showed a progressive consumption of both comonomers (Fig. 2e and S27), with ODP reaching nearly quantitative conversion after 28 h, when the consumption of VAc was 70–90% (Table 1). Importantly, SEC analysis showed the progressive increase of *M*_n_ with total monomer conversion (Fig. 2f), whereas P(VAc-*co*-ODP) exhibited *M*_n_ values closely approaching theoretical molar masses (Fig. S28 and Table 1), with *F*_ODP_ = 4 and 32 mol% (Fig. S29–S31), and much lower *Đ* ∼ 1.5–1.8 compared to the corresponding copolymers synthesized by FRP.

We further investigated the photoiniferter RAFT/MADIX copolymerization of ODP and DEGVE, with [DEGVE]_0_ : [ODP]_0_ = 85 : 15 (Table 1). ODP was consumed approximately 5-fold faster than DEGVE (Fig. S32 and S33), resulting in the formation of a gradient copolymer with *F*_ODP_ = 18 mol% (Fig. S34).

**Fig. 3:**
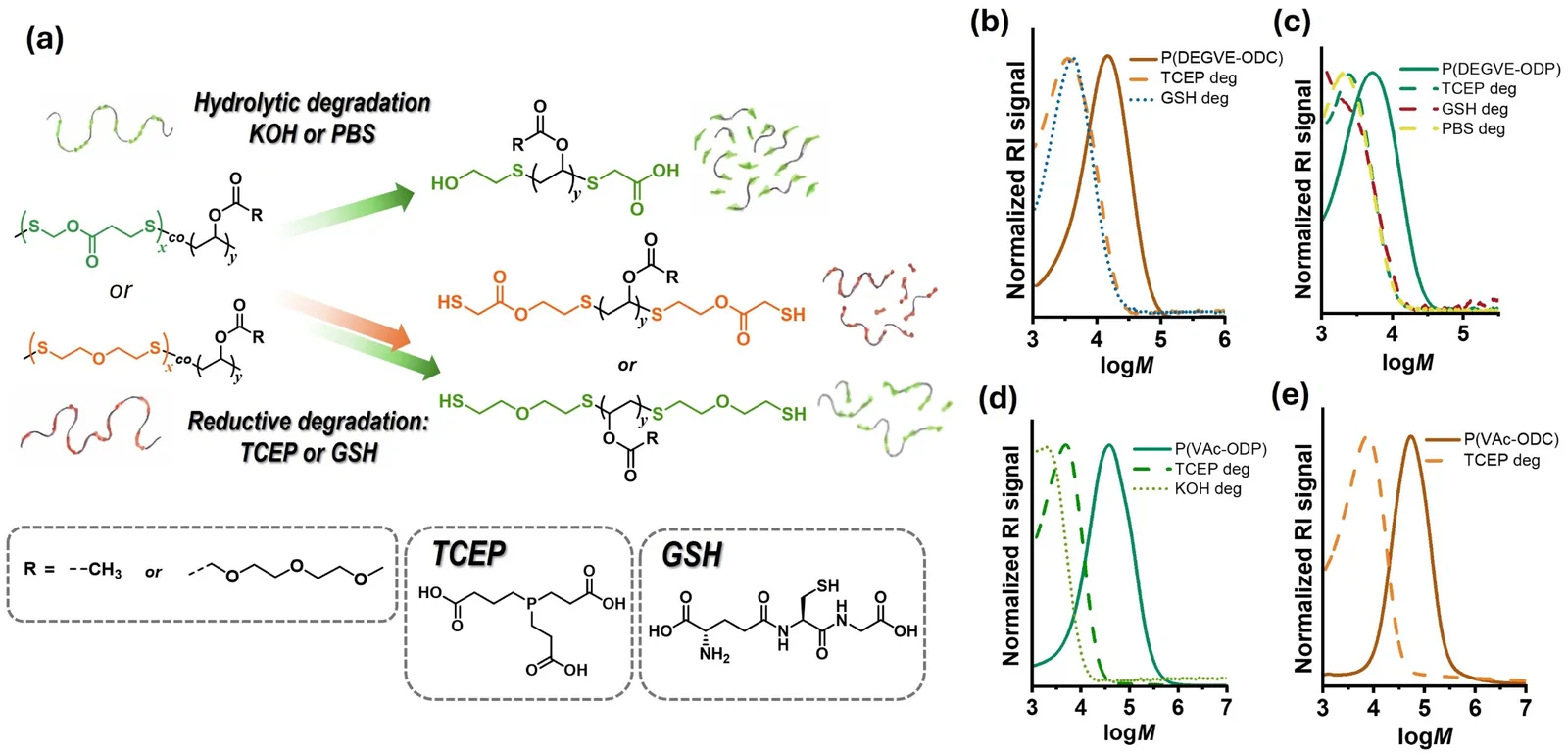
Degradation of polyvinyls incorporating ODP and ODC units. (a) Dual degradation pathways: disulfide bond cleavage using TCEP as mild reductant, or an aqueous solution of GSH to promote thiol-disulfide exchange for water-soluble copolymers; hydrolytic degradation of ester groups in ODP-based copolymers in KOH solutions or in PBS for water-soluble copolymers. GPC traces before and after degradation of (b) P(DEGVE-*co*-ODC), *F*_ODC_ = 24 mol%, (c) P(DEGVE-*co*-ODP), *F*_ODP_ = 18 mol%, (d) P(VAc-*co*-ODP), *F*_ODP_ = 22 mol%, (e) P(VAc-*co*-ODC), *F*_ODC_ = 13 mol%.

The incorporation of disulfide and ester functionalities within the polymer backbone gives access to multiple degradation pathways (Fig. 3a). To investigate these behaviors, the degradation of the polyvinyl copolymers was evaluated under both reductive and hydrolytic conditions. Cleavage of disulfide linkages was first examined using tris(2-carboxyethyl)phosphine (TCEP) as a reducing agent, while GSH was employed to probe degradation mediated by thiol-disulfide exchange under biologically relevant conditions.^31–33^ In parallel, ODP-containing copolymers were subjected to hydrolytic treatments in phosphate-buffered saline (PBS) and KOH solutions to assess the susceptibility of the ester-containing backbone to hydrolysis, thereby providing preliminary insight into their potential biodegradability.

Both copolymers including ODC and ODP present disulfide linkages along their backbone at ODC–ODC and ODP–ODP diads, this enabled fast and efficient reductive degradation when subjected to TCEP solutions (Fig. S35–S39). Incubation in THF/H_2_O solution of TCEP of P(VAc-*co*-ODC) with *F*_ODC_ = 13 mol% resulted in an 88% decrease in molar mass, reaching *M*_n_ = 3.6 kg mol^−1^ (Table 2). When *F*_ODC_ was increased to 32 mol% the same copolymer type degraded even more extensively (94% reduction of *M*_n_), providing polymer fragments with *M*_n_ = 1.4 kg mol^−1^. Under similar conditions, P(DEGVE-*co*-ODC) with *F*_ODC_ = 19 mol% was degraded by ∼70% yielding segments with *M*_n_ = 3.8 kg mol^−1^ (Table 2). The same water-soluble copolymer could be also degraded by incubation in 0.01 M solution of GSH, which led to a similar chain fragmentation, reaching *M*_n_ = 3.2 kg mol^−1^ (Fig. 3b).

**Table 2:** Degradation of representative copolymers of cyclic disulfide (*M*_S–S_) and vinyl ester (*M*_vin_) monomers^a^

Entry	*M*_S–S_	*M*_vin_	*F*_ODC or ODP_ b (mol%)	*M*_n_ c (kg mol^−1^)	*Đ* c	*M*_n,deg TCEP_ c (kg mol^−1^)	*M*_n,deg KOH_ c (kg mol^−1^)	*M*_n,deg GSH_ d (kg mol^−1^)	*M*_n,deg PBS_ d (kg mol^−1^)
1	—	VAc	0	51.0	6.2	51.0	n.d.	—	—
2	—	DEGVE	0	24.0	6.1	—	n.d.	23.8	23.9
3	ODC	VAc	13	30.3	1.94	3.6	—	—	—
4		VAc	32	22.2	2.23	1.4	—	—	—
5		DEGVE	24	12.4	1.70	3.4	—	3.2	—
6	ODP	VAc	8	28.6	2.55	14.5	—	—	—
7		VAc	22	14.3	3.79	4.0	2.0	—	—
8		DEGVE	18	4.5	1.85	2.1	1.8	2.4	2.5

a Degradation were performed using: (i) TCEP in THF/H_2_O 4/1 v/v to reduce disulfide bonds; (ii) hydrolytically in a solution of 0.1 M KOH in THF/MeOH 3/1 v/v for 90 min; (iii) an aqueous solution of 0.01 M GSH; (iv) an aqueous solution of PBS (pH = 7.4) for 3 days at *T* = 37 °C.

b Determined by ^1^H NMR on the purified copolymers.

c Measured by SEC using THF as eluent for *M*_vin_ = VAc, or DMF (with 1 g L^−1^ LiBr) as eluent for *M*_vin_ = DEGVE.

d Measured by SEC using DMF (with 1 g L^−1^ LiBr) as eluent.

In a similar manner, P(VAc-*co*-ODP) and P(DEGVE-*co*-ODP) were efficiently degraded upon treatment with TCEP, exhibiting decreases in molar mass ranging from 50 to 70% as a result of disulfide bond reduction (Table 2, Fig. 3c and d).

Particular attention was devoted to the multiple degradation pathways accessible to P(DEGVE-*co*-ODP), which displayed an initial *M*_n_ of 4.5 kg mol^−1^. This copolymer underwent efficient degradation not only upon TCEP-mediated disulfide reduction and GSH-mediated thiol-disulfide exchange, but also through hydrolytic backbone fragmentation under basic conditions in aqueous KOH and after incubation in PBS for 3 days (Fig. S40 and S41). Treatment with either TCEP or GSH induced cleavage of disulfide linkages, resulting in an approximately 50% decrease in *M*_n_ in both cases (Table 2 and Fig. 3c). A comparable reduction in molar mass was also observed following hydrolysis of the ester functionalities derived from ODP units along the polyvinyl backbone. These results demonstrated that such water-soluble poly(vinyl ester)s can undergo substantial degradation through distinct and orthogonal chemical pathways.

Having demonstrated that ODP-containing polyvinyls can undergo degradation through multiple pathways—including disulfide cleavage and ester hydrolysis—we next sought to evaluate how these distinctive features could coexist with the functional properties required in technologically relevant applications.

In particular, we focused on P(DEGVE-*co*-ODP) copolymers, which are water-soluble and incorporate OEG side chains. Non-degradable, OEG-bearing poly(meth)acrylates have been emerging as relevant polymers within the biomaterials field because they retain the bioinertness of polyethylene glycol (PEG) while exhibiting significantly reduced immunogenicity.^34–38^ Structurally related poly(vinyl ester)s were initially introduced by O'Reilly and Dove,^26^ primarily to investigate their thermoresponsive behavior in aqueous media, whereas their interactions with biological environments have only very recently begun to be explored.^27^

More generally, the design of water-soluble polyvinyls that combine bioinertness with hydrolytic degradability would meet the need for safe, non-toxic performance during use while ensuring controlled degradation when they enter the environment.^39–42^ This would enable the generation of sustainable alternatives to persistent PEGs or polyvinyl pyrrolidone (PVP) not only for key applications in biomedicine, but also in the development of polymer formulations for personal/home care products (*e.g.*, cosmetics and detergents) and agriculture.

**Fig. 4:**
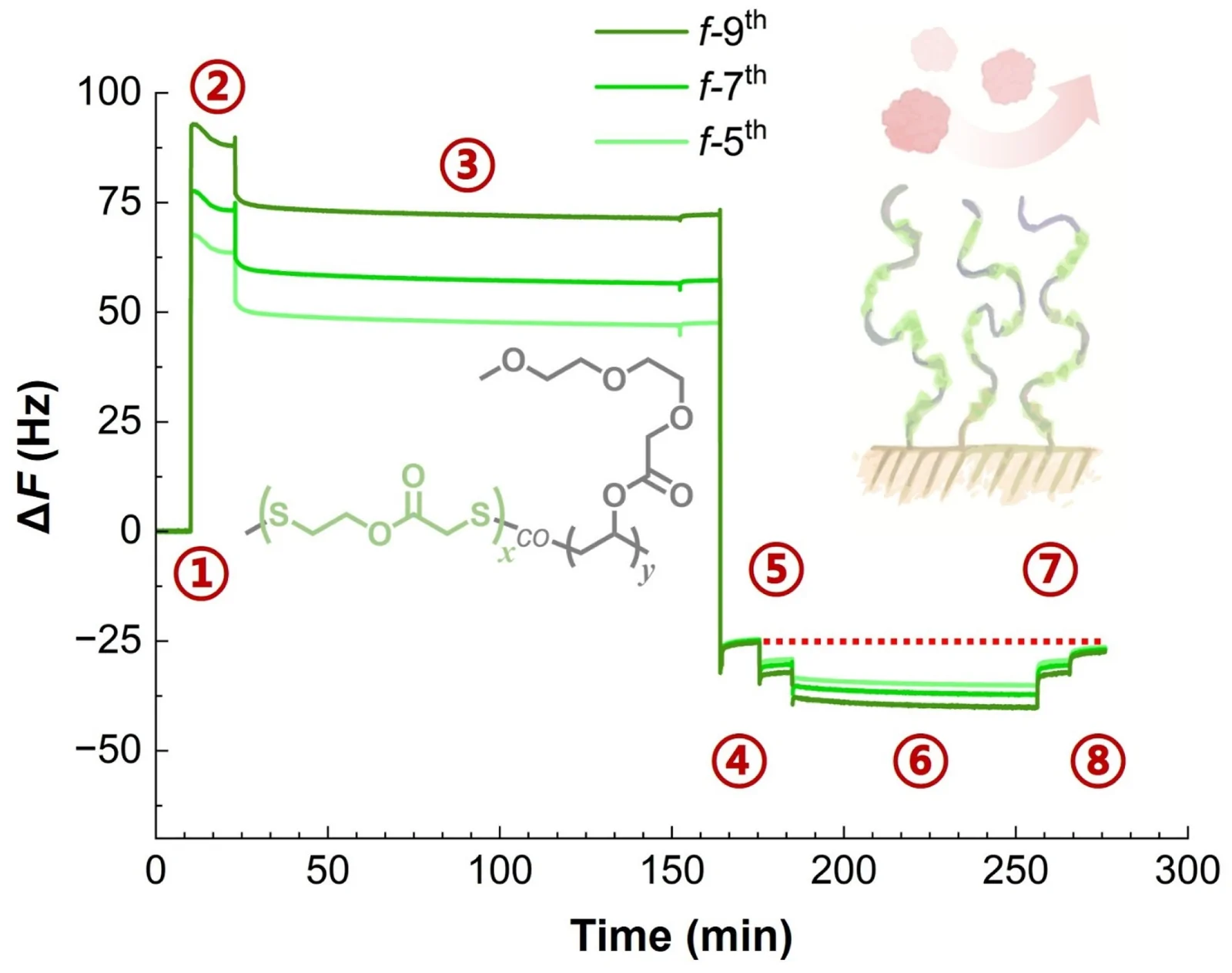
QCMD sensograms reporting the variation of Δ*F* during the assembly of P(DEGVE-*co*-ODP) brushes on Au-coated sensors and upon interaction with protein-rich media. Three different overtones (f-5th, f-7th and f-9th) were reported for each sample. Point (1) corresponds to the injection of ultrapure water, point (2) corresponds to flushing methanol. Then, at point (3) a solution of copolymer in methanol was injected, followed by rinsing with methanol. At point (4) ultrapure was injected followed by PBS buffer at point (5). Then, a solution of 10% human serum was injected at point (6), followed by rinsing with PBS (7), and ultrapure water (8). The recovery of approximately the same frequency recorded prior to the injection of 10% human serum indicates negligible interaction of the brushes with proteins.

In order to test their interaction with protein-rich media P(DEGVE-*co*-ODP) copolymers with *F*_ODP_ = 14 mol% and 38 mol%, and *M*_n_ = 8.9 and 6.5 kg mol^−1^ (Table S2) were first assembled on Au substrates—by exploiting both the xanthate fragments and the disulfide/thioether moieties as anchoring groups with high affinity towards Au^43^—and subsequently incubated in 10% human serum. “Grafting-to” of P(DEGVE-*co*-ODP) copolymers on Au provided brush layers with dry thickness (*T*_dry_) ∼ 0.85 nm, as determined by variable angle spectroscopic ellipsometry (VASE), corresponding to copolymer grafting densities (*σ*) of 0.06–0.09 chain nm^−2^. The comonomer composition had no substantial influence on *T*_dry_. Quartz crystal microbalance with dissipation (QCMD) was then employed to determine the properties of P(DEGVE-*co*-ODP) brushes in the swollen state (Fig. 4). The values of swollen thickness (*T*_wet_) were obtained by fitting the variation in frequency (Δ*F*) and dissipation (Δ*D*) with an extended viscoelastic model,^44^ giving *T*_wet_ = 4.7 ± 0.2 and 4.0 ± 0.1 nm for the copolymer with 14 mol% and 38 mol% ODP, respectively. From the values of *T*_dry_ and *T*_wet_, the water content was calculated as *W*% = [(*T*_wet_ − *T*_dry_)/*T*_wet_] × 100 = 85% (14 mol% ODP) and 80% (38 mol% ODP). Thus, P(DEGVE-*co*-ODP) copolymers with different composition displayed similar *T*_dry_ and *σ* values—which were also close to the values recently obtained for PDEGVE homopolymers assembled on identical Au surfaces^27^—but different swelling properties, as higher molar fraction of DEGVE determined higher swelling of the brushes. The brush-coated sensors were then exposed to 10% human serum for 1 h and then rinsed with PBS. Remarkably, a negligible amount of adsorbed serum proteins was recorded for both films (Fig. 4) demonstrating the bioinert character of P(DEGVE-*co*-ODP) species when assembled on solid substrates to form brush layers.

## Conclusions

Functional seven-membered cyclic disulfides emerge as a versatile platform for the synthesis of degradable polyvinyl materials through radical copolymerization. While ODC enabled the incorporation of disulfide functionalities within polyvinyl ester backbones, ODP additionally introduced hydrolysable ester groups, thereby providing access to polymers susceptible to multiple and orthogonal degradation pathways.

Both free radical polymerization and photoiniferter RAFT/MADIX polymerization proved effective for the synthesis of these copolymers, enabling incorporation of cyclic disulfide units within polyvinyl esters, including water-soluble materials based on DEGVE. The resulting copolymers underwent efficient degradation through reductive disulfide cleavage, GSH-mediated thiol-disulfide exchange, and hydrolytic fragmentation under mildly basic or physiological conditions. Furthermore, surface-assembled P(DEGVE-*co*-ODP) copolymers formed highly hydrated and protein-repellent interfaces, demonstrating that bioinert interfacial properties can coexist with multi-pathway degradation.

Overall, these results highlight how the rational molecular design of cyclic disulfides enables the synthesis of polyvinyl copolymers that are not only redox-degradable but also hydrolysable and therefore potentially biodegradable under environmentally relevant conditions. By extending this concept to water-soluble and bioinert architectures, this work paves the way toward a new generation of sustainable polymeric materials with strong potential for translation into biomedical, consumer-care, and advanced materials applications.

## Author contributions

The manuscript was written through contributions of all authors. All authors have given approval to the final version of the manuscript.

## Conflicts of interest

There are no conflicts to declare.

## Supplementary Material

SC-OLF-D6SC04278C-s001

## Data Availability

Materials and experimental methods, NMR and SEC analysis, additional kinetics data can be found in the supplementary information (SI). Supplementary information is available. See DOI: https://doi.org/10.1039/d6sc04278c.
