# Supplementary material for: Functional cyclic disulfides enable multi-pathway degradable polyvinyls
Source: Chem Sci. 2026 Aug 25;17(40):19325–32. doi: 10.1039/d6sc04278c (PMC13523016; doi:10.1039/d6sc04278c)
Supplement: Supplementary file 1 [file SC-017-D6SC04278C-s001.pdf]

Supplementary Information (SI) for Chemical Science.
This journal is © The Royal Society of Chemistry 2026

SUPPORTING INFORMATION

Functional Cyclic Disulfides Enable Multi-Pathway Degradable Polyvinyls

Alessandro Magro,[a] Elena Avanzini, [a] Daniele Rizzi, [a] Giovanni Gobbo, [a] Francesco M. Ara,[a] Rachele Foladore, [a] Alessandro Bertolo,[a] Cristian Pezzato,[a] Gianluca Gazzola,[b] Francesca Lorandi,*[a] Edmondo M. Benetti*[a]

[a] A. Magro, E. Avanzini, D. Rizzi, G. Gobbo, F.M. Ara, R. Foladore, A. Bertolo, Prof. C. Pezzato, Dr. F. Lorandi, Prof. E.M. Benetti
Laboratory for Macromolecular and Organic Chemistry (MOC), Department of Chemical Sciences
University of Padova
via Marzolo 1, 35131 Padova, Italy
E-mail: francesca.lorandi@unipd.it, edmondo.benetti@unipd.it

[b] Dr. G. Gazzola
Amethyst Lab SRL
via Tiberio Deciani 2, 33100 Udine, Italy

Materials

All reagents, materials, and solvents employed in the syntheses were purchased from commercial suppliers and used as received, without further purification. For the polymerization, commercial vinyl monomers were passed through activated basic alumina (Al_2O_3), Brockmann I (Sigma-Aldrich).

Bis(2-mercaptoethyl) ether (>95%, TCI); Iodine (>99%, TCI); Triethylamine (>99%, Merck); Sodium thiosulfate pentahydrate (>99%, TCI); 2-Mercaptoacetic acid (>95%, TCI); 2-Mercaptoethanol (>99%, TCI); p-Toluenesulfonic acid (>98%, TCI); Sodium bicarbonate (>95%, Merck); Sodium sulfate anhydrous (>99%, Merck); Sodium iodide (>99%, TCI); Hydrogen peroxide (>99%, Merck); Potassium hydroxide (>99%, Merck); [2-(2-methoxyethoxy)ethoxy]acetic acid (>95%, Merck); $Pd(OAc)_2$ (>99%, Merck); Vinyl acetate (>99%, Merck); Methyl 2-bromopropanoate (>95%, Merck); Potassium O-ethyl dithiocarbonate (>95%, TCI); 2,2′-azobis(2-methylpropionitrile) (>99%, Merck). All solvents are purchased from Merck or Carlo Erba.

Deuterated solvents used for the acquisition of ^{1}H and ^{13}C NMR spectra were purchased from commercial suppliers (Sigma-Aldrich, Eurisotop) and possessed a minimum isotopic purity of 99.8%.

Thin-layer chromatography (TLC) analyses were performed on silica gel 60 F254 aluminum sheets (Merck). Kieselgel 60M silica (0.063–0.40 mm) was used as the stationary phase for column (flash) chromatography. Visualization of the developed chromatograms was carried out by checking UV absorbance at $\lambda = 254$ nm as well as by staining with an aqueous potassium permanganate solution.

MilliQ water was purified with a Millipore Direct-Q5 ultrapure water system or a Milli-Q IQ 7003 purification system.

Instruments

Nuclear magnetic resonance (NMR) spectroscopy. ^{1}H NMR and ^{13}C NMR spectra were recorded using a Bruker AVANCE NANOBAY spectrometer at 400 MHz and equipped with a Varian 400/54

(9.39 T) magnet and a BBFO probe. Chemical shifts (δ) are given in ppm relative to the signal of the residual non-deuterated solvent.

DOSY 2D maps were conducted using a Bruker AVII400 Ultra-Shield spectrometer operating at 400 MHz were obtained by dstebpgp3s pulse sequence. Each spectrum was processed with both Topspin 3.1 and Dynamics Center 2.2.1 (Bruker BioSpin GmbH, 2014). Diffusion coefficients were extracted from fits of the peak intensities versus gradient strengths using the equation:

$$I_G = I_{G0}(-(\gamma\delta G)^2 D\left(\Delta - \frac{\delta}{3}\right))$$

where I_{G0} is the signal intensity in the absence of the gradient spin-echo, G the gradient strength, D the diffusion coefficient, Δ the diffusion delay, δ the length of the gradient pulses and γ the magnetogyric ratio of the observed nuclide

Size exclusion chromatography (SEC). For homopolymers and copolymers containing vinyl acetate, SEC was performed using a Shimadzu NEXERA system equipped with two PSS Agilent columns and one Phenogel column (SDV, 300 × 8 mm, pore sizes of 500, 1000, and 10^5 Å), and a refractive index detector. Stabilized tetrahydrofuran (THF) was used as the mobile phase at a flow rate of 1 mL min^{-1} and a temperature of 40 °C. For homopolymers and copolymers containing ethylene oxide units in the side chains, SEC was performed using a Viscotek 302-TDA instrument equipped with a refractive index (RI) detector, a GRAM pre-colum (50x8 mm) and two GRAM analytical linear columns (300x8 mm) from Agilent PSS connected in series. The column compartment and RI detector were both at 60 °C, and the eluent used was *N,N*-dimethylformamide (DMF + LiBr 1g/L). Instruments' calibration was performed using poly(methyl methacrylate) (PMMA) standards of known molecular weight (2680–400000 Da) supplied by Polymer Laboratories Ltd.

Mass spectrometry. Mass spectrometric analyses were performed using an Agilent 5975 MSD mass spectrometer coupled to an Agilent 6850 gas chromatograph. Helium (He) was employed as the carrier gas. The GC system was equipped with a split injector (interface temperature: 250 °C) and an Agilent 19091S-433E capillary column (HP-5MS stationary phase, 5% phenyl-methyl-polysiloxane; 30 m length, 250 μm internal diameter, 0.25 μm film thickness). The Agilent 5975 MSD, operated with an autosampler, was equipped with an electron-impact (EI) ionization source (70 eV; source temperature: 230 °C), a scanning quadrupole analyzer, and an EMD detector.

Quartz crystal microbalance with dissipation (QCM-D). The assembly of polymer adsorbates on Au surfaces and interaction with proteins were monitored in situ by QCM-D, using an E4 instrument (Q-Sense AB, Göteborg, Sweden) equipped with dedicated Q-Sense AB software. Au-coated crystals (Biolin Scientific) with a fundamental resonance frequency of 4.95 MHz were used as substrates. Before the experiment, the substrates were cleaned in a UV-cleaner (Boekel Industries, Inc.), immersed in a piranha solution (H_2SO_4:H_2O_2 3:1) for 30 seconds, rinsed with ultrapure water, EtOH and dried under a stream of N_2. Then, they were exposed to ultrapure water at ambient temperature until a stable baseline was recorded. Later, ultrapure water was replaced with MeOH, followed by the injection of a 1 mg mL^{-1} polymer solution in MeOH until complete adsorption was attained (2h). To eliminate the physiosorbed polymer adsorbates, washing steps were performed with MeOH and ultrapure water. A second cycle of injections was performed to evaluate the interaction between the polymer layer and human serum. Ultrapure water was substituted with phosphate buffered saline (PBS, 10 mM), followed by lyophilized human serum in PBS. Later, the substrates were rinsed to remove weakly bound proteins.

The values of hydrated thickness (T_{wet}) of the polymer layer were obtained by applying an extended viscoelastic model, fitting the frequency shifts (ΔF), by using three different overtones (fifth, seventh, and ninth) for each sample. Three crystals for each film were used to calculate the mean values of T_{wet} and their standard deviations. The input parameters for the fitting were fluid density (997 kg

m^{-3}), layer density (1100 kg m^{-3}) and fluid viscosity (0.009 kg m^{-1} s^{-1}). The following parameters were fitted while constrained to physically meaningful boundaries: layer viscosity (0.009−0.05 kg m^{-1} s^{-1}), layer shear thickness (104−108 Pa), and layer thickness ($10^{-9} - 2 \times 10^{-8}$ m). The values of hydrated protein layer ($T_{protein}$) were obtained by applying a similar fitting procedure.

Variable angle spectroscopic ellipsometry (VASE). The values of dry thickness (T_{dry}) of the polymer adsorbates on gold substrates were measured with a variable-angle spectroscopic ellipsometer (M-2000F, Woollam Co., Inc., Lincoln, NE). The measurements were recorded within a range of wavelengths included between 400 and 850 nm using focusing lenses at 65° from the surface normal. Each data point resulted from an average of 20 measurements, and the obtained raw data were fitted with a bilayer model (Au and organic layer) using the analysis software WVASE32. The optical constants of the Au substrate (n and k) were experimentally obtained by measuring a freshly cleaned Au substrate with known thickness (200 nm). The organic adlayer were fitted using a Cauchy model approximated to $n = A + B\lambda^{-2}$ where n is the refractive index, λ is the wavelength, and A and B were assumed to be 1.45 and 0.01, respectively. The values of T_{dry} were used to calculate the grafting density (σ) by applying the equation $\sigma = \rho T_{dry} N_A M_n^{-1}$, where ρ is the density of the dry polymer, N_A is the Avogadro number, and M_n is the number-average molecular weight of the adsorbate. For all VASE experiments, mean values and standard deviations were calculated using four replicates per sample and measuring five points on each of them.

Procedures

General procedure for thermal homopolymerization of ODC and ODP. The desired amounts of monomers and AIBN (2 mol%) were introduced into a Schlenk flask sealed with a rubber septum and cooled in an ice bath. For the polymerization in solution of ODC, toluene was added to reach a concentration $[ODC]_0$ = 3M. The reaction mixture was degassed with Argon for 20 minutes at 0 °C and subsequently placed in a preheated oil bath at 80 °C. Polymerization kinetics were monitored by

periodically withdrawing small aliquots of the reaction mixture, diluting them in $CDCl_3$, and measuring the relative decrease of the monomer resonances by ^1H NMR spectroscopy.

For the purification of PODC, the crude reaction mixture was diluted with dichloromethane and precipitated twice in a 9:1 v/v $CH_3OH:H_2O$ mixture, followed by two additional precipitations in *n*-hexane. The precipitated polymer was recovered by centrifugation and the solvents removed under reduced pressure.

The purification of PODP was carried out following the same procedure, precipitating the crude reaction mixture in methanol.

General procedure for thermal copolymerization of ODC or ODP with vinyl monomers. The desired amount of monomers, AIBN (0.5 mol% and 2 mol%), hexadimethylsiloxane (HDMSO, 10 mol% as internal standard) and toluene as solvent (to have a total monomer concentration of 3 M, except for vinyl esters that were polymerized in bulk) were placed in a Schlenk flask sealed with a rubber septum. The mixture was cooled in an ice bath and degassed with argon for 20 min, then transferred to a preheated oil bath at 70 °C. The polymerization kinetics were monitored by sampling small aliquots of the reaction mixture over time, diluting them in $CDCl_3$, and measuring the relative decrease of monomer signals by ^1H NMR spectroscopy.

The crude reaction mixture was diluted with the minimum amount of dichloromethane and precipitated twice in cold *n*-hexane. The polymer was collected by centrifugation followed by removal of the liquid phase. Residual solvents were removed under reduced pressure.

General procedure for copolymerization of ODC or ODP with vinyl esters via photoiniferter. The desired amount of monomers, 2-(methyl propionate)-O-ethyl xanthate and or as CTA, and HDMSO (10 mol% as internal standard) were added to a vial sealed with a rubber septum. The mixture was cooled in an ice bath and degassed with argon for 30 min. The polymerization mixture was then irradiated with a Kessil lamp placed approximately 3.5 cm from the vial ($\lambda_{max} = 427$ nm, 25%

intensity corresponding to ~70 mW cm^{-2}). During irradiation, a fan was oriented toward the setup to maintain the polymerization mixture at room temperature. The copolymers were purified via precipitation in cold *n*-hexane.

Procedure for FRP of vinyl acetate (VAc) in the presence of PODP. The desired amount of VAc, AIBN (2 mol%), hexadimethylsiloxane (HDMSO, 10 mol% as internal standard) and PODP ($[VAc]_0$:$[ODP]_0$ = 85:15) were placed in a Schlenk flask sealed with a rubber septum. The mixture was cooled in an ice bath and degassed with argon for 20 min, then transferred to a preheated oil bath at 70 °C. The polymerization kinetics were monitored by sampling small aliquots of the reaction mixture over time, diluting them in $CDCl_3$, and measuring the relative decrease of monomer signals by ^{1}H NMR spectroscopy.

General procedure for copolymer degradation with TCEP. In a vial, 50 mg of the copolymer was dissolved in a minimal volume of THF. A solution of TCEP (5 eq. relative to the incorporated $M_{s\text{-}s}$ units) in THF/water (4:1 v/v) was then added, and the reaction mixture was stirred at room temperature for 24 h. The solvents were removed under reduced pressure, after which DCM was added to the residue. The resulting mixture was washed sequentially with aqueous $NaHCO_3$ and brine. Solvents were removed under reduced pressure to obtain the degradation products.

General procedure for copolymer degradation with KOH. A stirred solution of the copolymer (100 mg) in THF (8 mL) was treated with a dropwise addition of a solution of KOH (0.5 wt% in 1.25 mL of methanol). The reaction mixture was stirred at room temperature for 1.5 h. Subsequently, the mixture was neutralized with dilute HCl and solvents were removed to dryness under reduced pressure. The resulting degradation products were characterized by ^{1}H NMR and SEC.

General procedure for copolymer degradation with GSH. In a vial, 5 mg of the copolymer was dissolved in 2 mL of an aqueous solution of glutathione reduced (10 mM). The resulting mixture was stirred for 36 h at room temperature and then freeze-dried. The resulting degradation products were characterized by ^{1}H NMR and SEC.

General procedure for copolymer degradation in PBS. In a vial, 20 mg of the desired copolymer was dissolved in 2 mL of PBS (pH 7.4) and the aqueous solution was stirred for 3 days (150 rpm, 37 °C). The solvent was removed to dryness under freeze dryer. The resulting degradation products were characterized by SEC.

Synthesis of monomers and chain transfer agent (CTA)

Synthesis of 1,4,5-oxadithiacycloheptane (ODC)

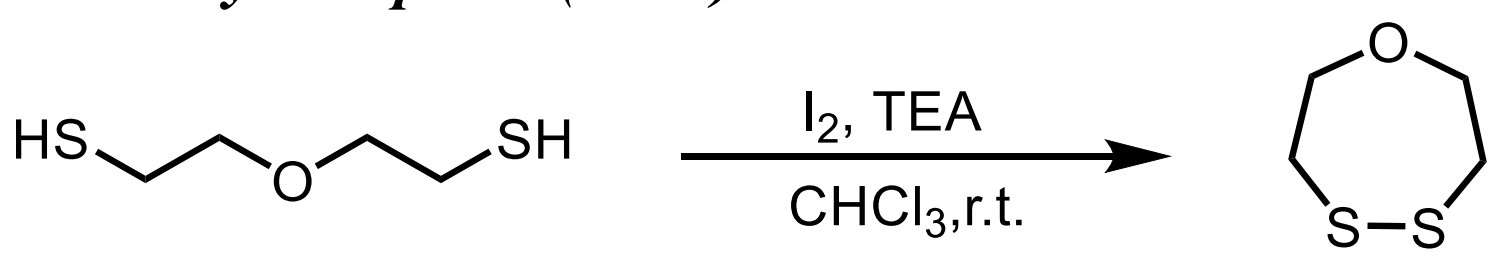

Bis(2-mercaptoethyl) ether (5.5 g, 40 mmol, 1.0 equiv) in $CHCl_3$ (100 mL, 0.4 M) and I_2 (10.1 g, 40 mmol, 1.0 equiv) in $CHCl_3$ (283 mL, 0.14 M) were added over 5 h to a stirred solution of triethylamine (11.1 mL, 80 mmol, 2.0 equiv) in $CHCl_3$ (307 mL, 0.26 M) at room temperature in a 3 L three-necked round-bottom flask. The reaction mixture was sequentially washed with 10% w/v $Na_2S_2O_3$ (2×), 1 N HCl (2×), and brine (2×). The organic layer was dried over Na_2SO_4, filtered, and concentrated under reduced pressure. The crude product was purified by vacuum distillation (~1.1 mbar, 80 °C) to give ODC as a colorless oil (3.836 g, 70% yield). **^{1}H-NMR** (400 MHz, $CDCl_3$): δ 4.11 (t, J = 5.4 Hz, 4H), 3.01 (t, J = 5.3 Hz, 4H). **^{13}C-NMR** (101 MHz, $CDCl_3$) δ 72.53, 41.11. **MS** (EI, 70 eV): m/z (relative intensity) calculated for $C_4H_8S_2O$: 136.00, found 136.000.

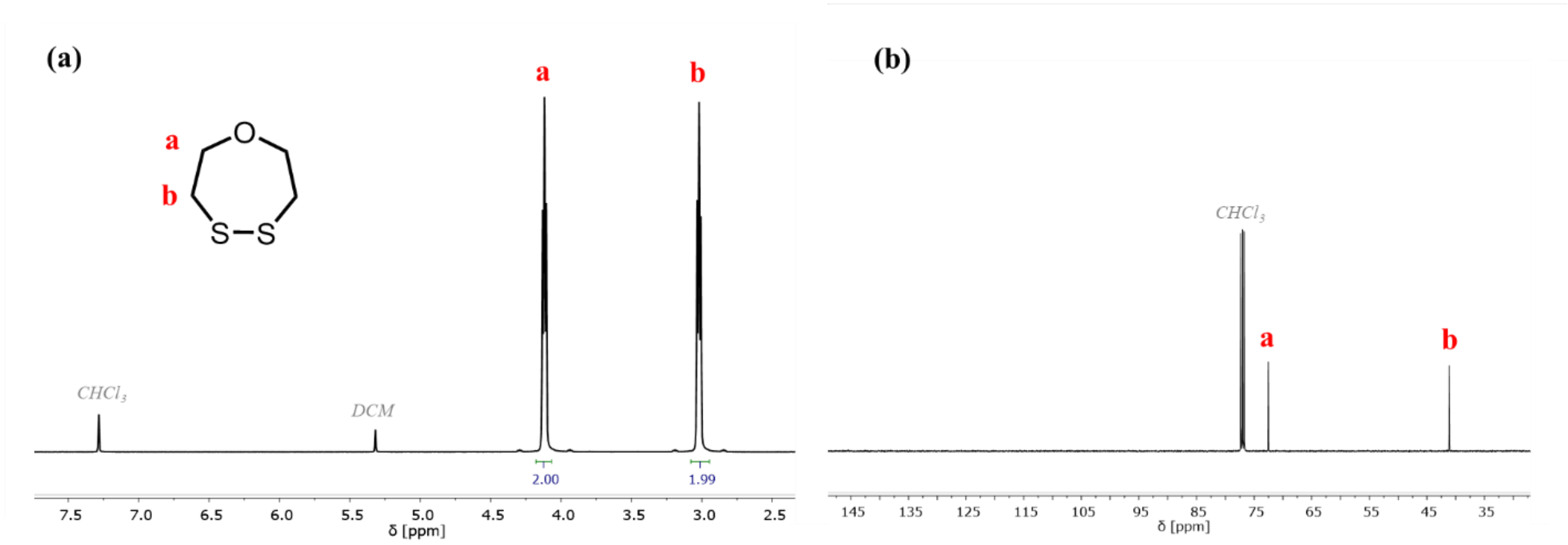

Figure S1. (a) ^{1}H NMR and (b) ^{13}C NMR spectra in $CDCl_3$ of ODC.

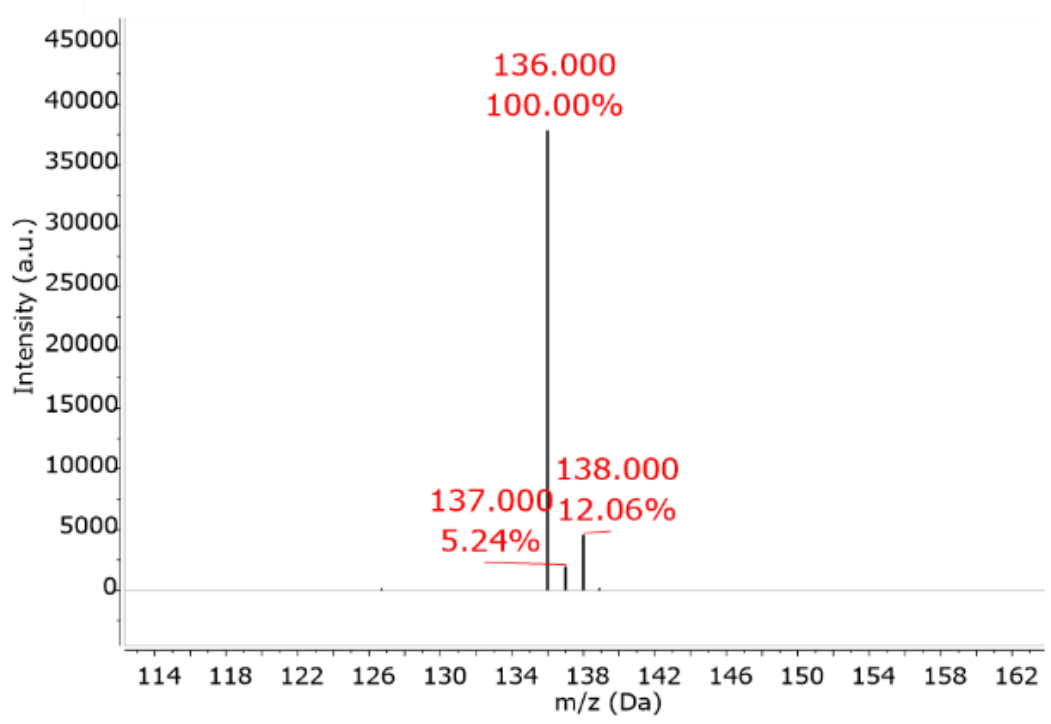

Figure S2. MS spectrum of ODC.

Synthesis of 1,4,5-oxadithiepan-2-one (ODP)

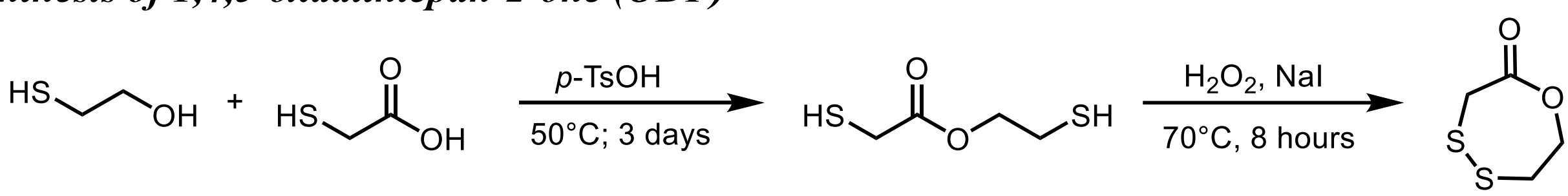

Synthesis of 2-mercaptoethyl 2-mercaptoacetate. 2-Mercaptoacetic acid (11.9 mL, 0.17 mol, 1.2 equiv) and p-toluenesulfonic acid (1.35 g, 7.1 mmol) were added to 2-mercaptoethanol (10 mL, 0.14 mol, 1.0 equiv) in a round-bottom flask. The mixture was sealed and stirred at 50 °C for 72 h. After the reaction, $CHCl_3$ (200 mL) was added, and the organic phase was washed twice with a mixture of distilled water : saturated $NaHCO_3$, 9:1 v/v. The organic layer was dried over anhydrous Na_2SO_4, filtered, and concentrated under reduced pressure. The crude product was purified by vacuum distillation (~1.1 mbar, 120 °C), yielding 2-mercaptoethyl 2-mercaptoacetate as a colorless oil (10.751 g, 50% yield). **^{1}H-NMR** (400 MHz, $CDCl_3$): δ 4.21 (t, J = 6.6 Hz, 2H), 3.25 (d, J = 8.4

Hz, 2H), 2.73 (t, J = 8.2 Hz, 2H), 2.01 (t, J = 8.4 Hz, 1H), 1.52 (t, J= 8.5 Hz, 1H). **^{13}C-NMR** (101 MHz, $CDCl_3$): δ 170.48, 66.65, 26.36, 23.11

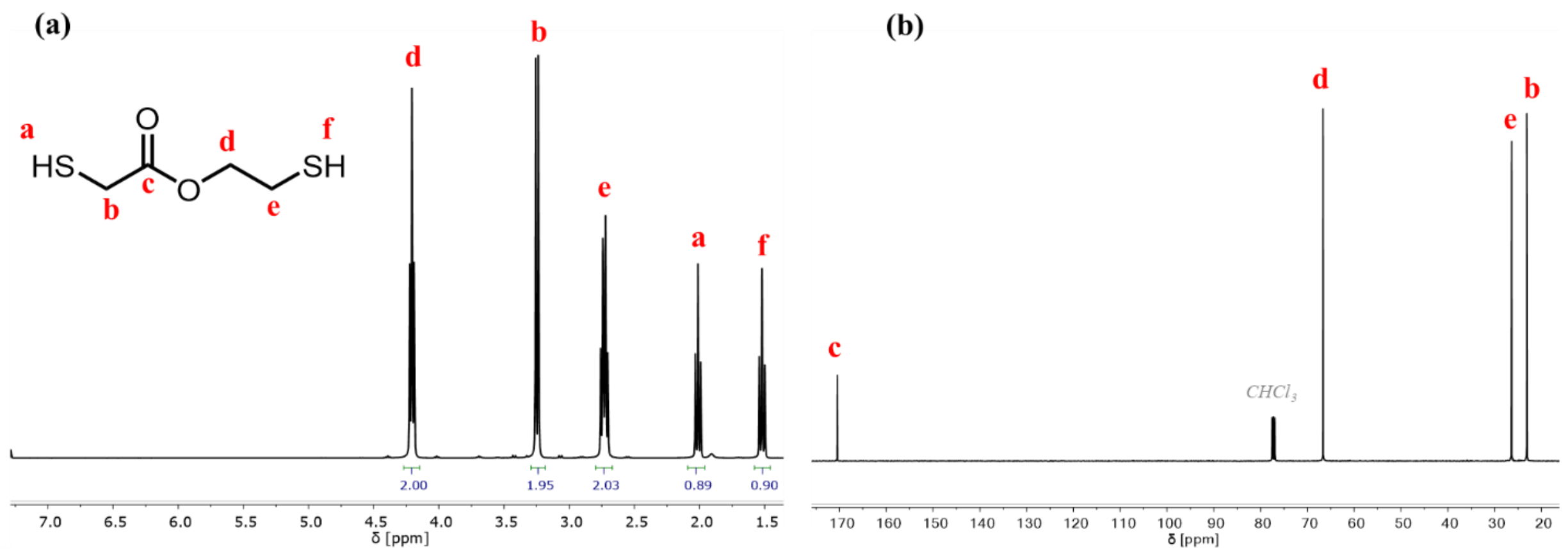

Figure S3. (a) ^{1}H NMR and (b) ^{13}C NMR spectra in $CDCl_3$ of 2-mercaptoethyl 2-mercaptoacetate.

Synthesis of 1,4,5-oxadithiepan-2-one (ODP). 2-mercaptoethyl 2-mercaptoacetate (1.520 g, 10.11 mmol, 1.0 equiv) was dissolved in EtOAc (200 mL) and added over 8 h under stirring to a solution of NaI (15 mg, 0.10 mmol, 0.01 equiv) and 30% w/w H_2O_2 (2 mL, 19.6 mmol, 1.96 equiv) in EtOAc (2 L) at 70 °C.

After the addition, the reaction mixture washed twice with aqueous $Na_2S_2O_3$ solution (~10% w/v) and twice with brine. The organic layer was dried over anhydrous Na_2SO_4, filtered, and concentrated under reduced pressure. The crude oil was purified by flash chromatography on silica gel (EtOAc : petroleum ether 2:8) to afford the product as a pale yellow oil (yield: 50%). **^{1}H-NMR** (400 MHz, $CDCl_3$): δ 4.65 (t, J = 4.9 Hz, 2H), 3.92 (s, 2H), 3.12 – 3.05 (m, 2H). **^{13}C-NMR** (101 MHz, $CDCl_3$): δ 169.25, 69.33, 41.94, 36.69. **MS** (EI, 70 eV): m/z (relative intensity) calculated for $C_4H_8S_2O_2$: 149.98, found:149.900.

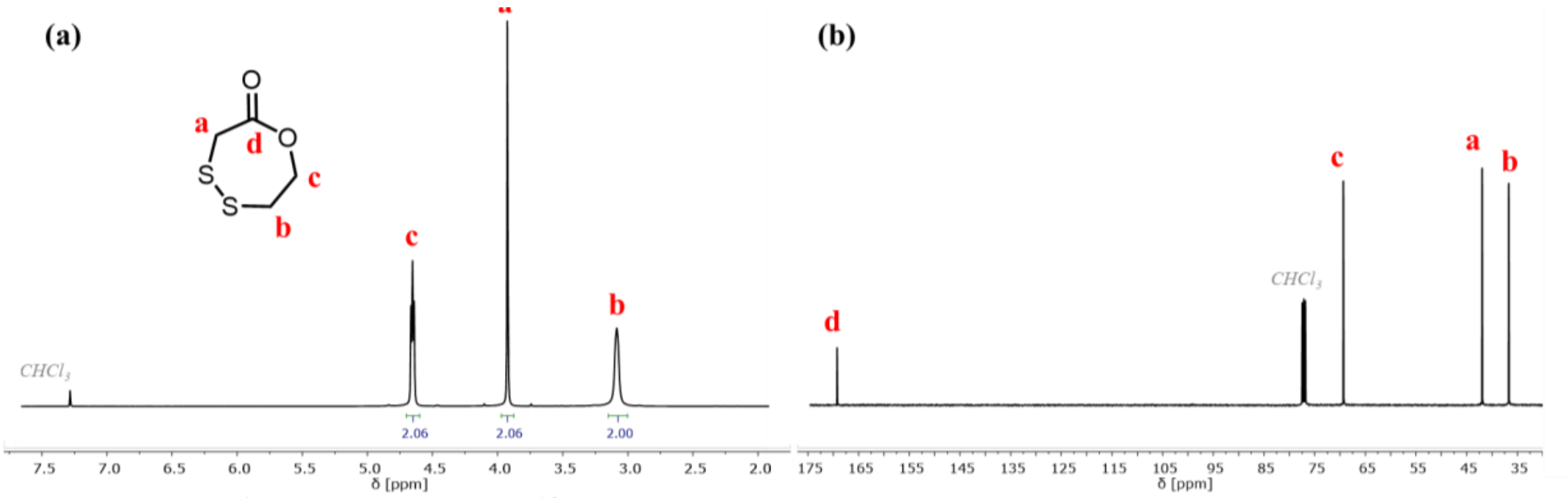

Figure S4. (a) ^{1}H NMR and (b) ^{13}C NMR spectra in $CDCl_3$ of ODP.

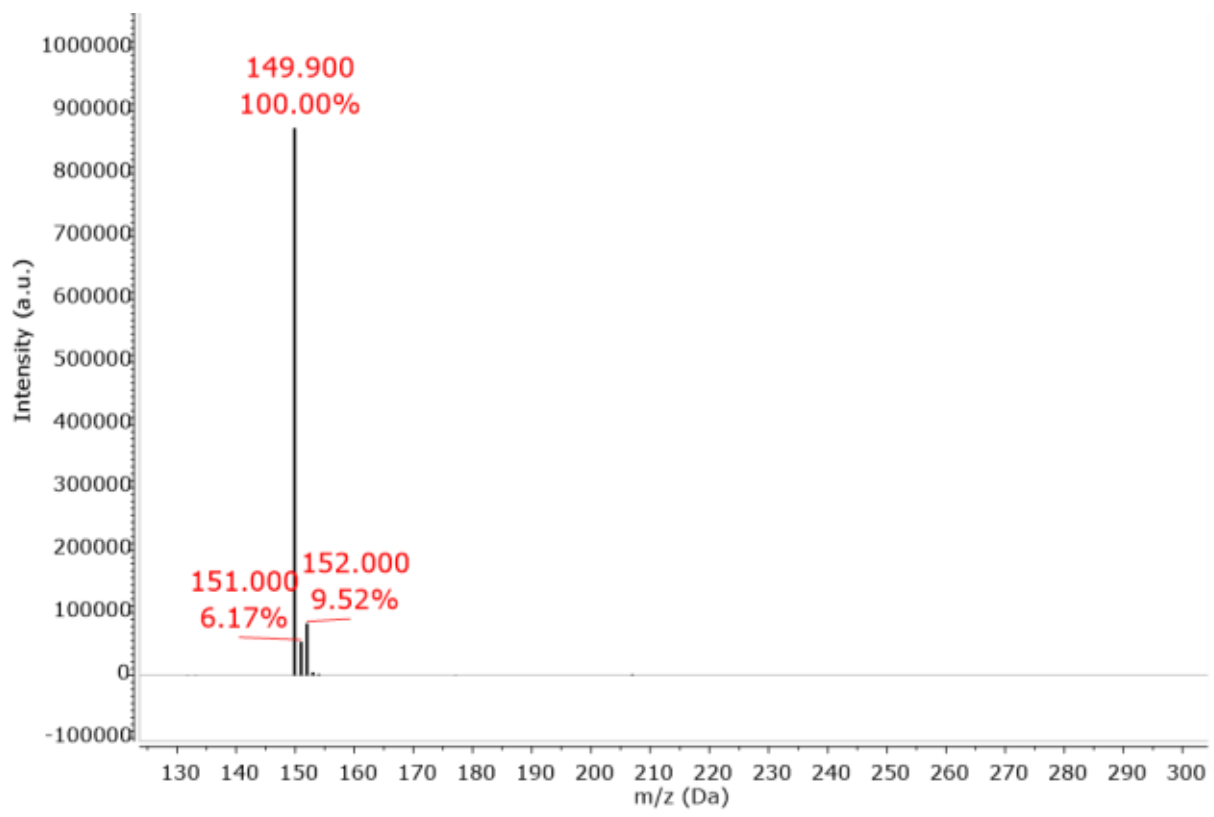

Figure S5. MS spectrum of ODP.

<u>Homopolymers of ODC and ODP</u>

Table S1. Homopolymerizations of ODC and ODP at 80 °C, using AIBN as radical initiator.[a]

Entry	$M_{S\text{-}S}$	*t* (h)	Conv. $M_{S\text{-}S}$ (%)[b]	M_n[c] (kg mol^{-1})	*Đ*[c]
1	ODC[d]	24	62	31.0	2.68
2	ODC	23	89	35.7	2.22
3	ODP	7	94	21.2	1.69

[a] (Co)polymerizations were performed in toluene, with a total monomer concentration of 3 M and 2 mol% AIBN relative to the monomer concentration. [b] Determined by ^{1}H NMR. [c] Measured by SEC using THF as eluent. [d] In bulk.

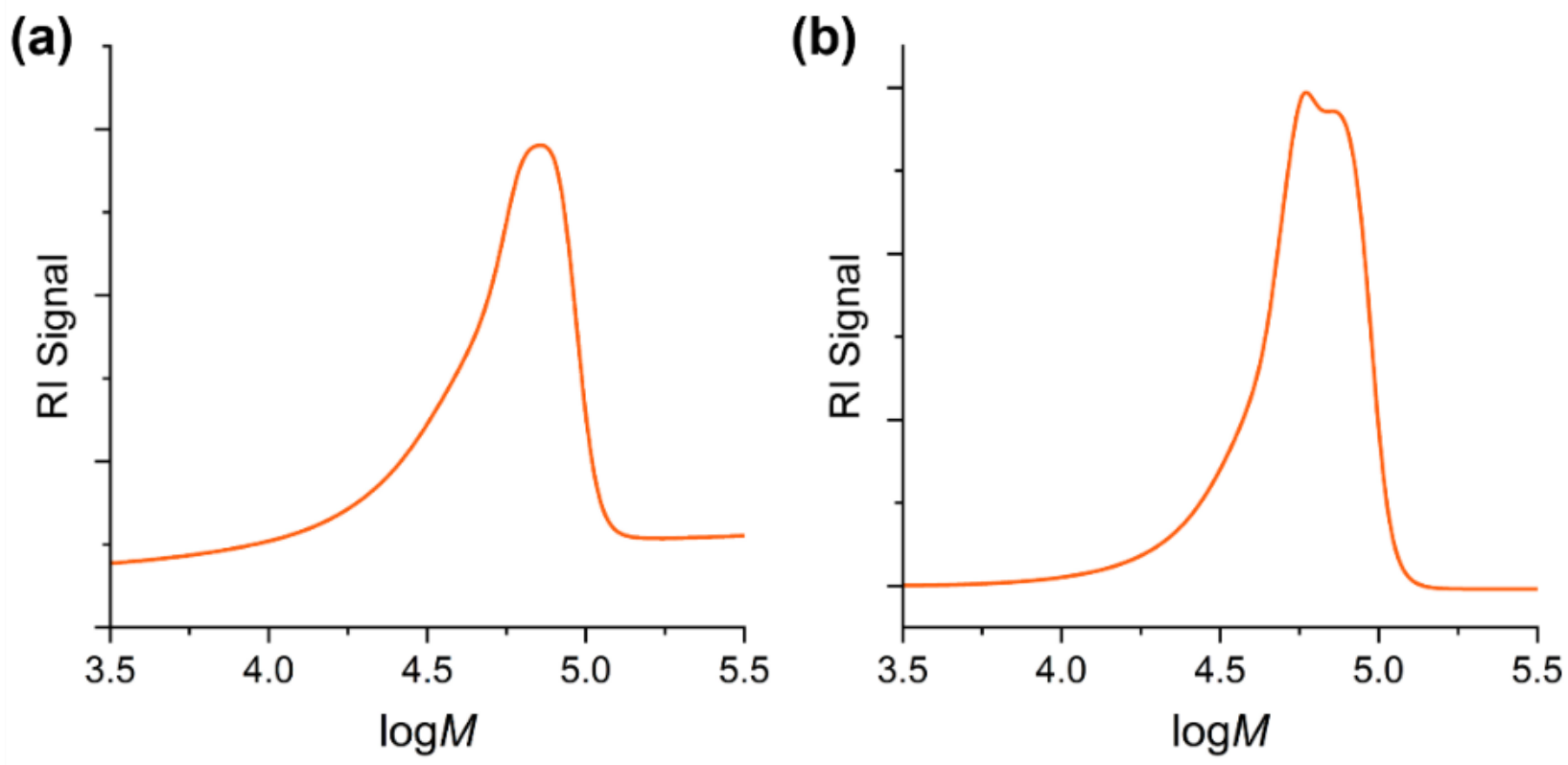

Figure S6. SEC traces of PODC via rROP in (a) bulk, (b) toluene ([ODC] = 3M).

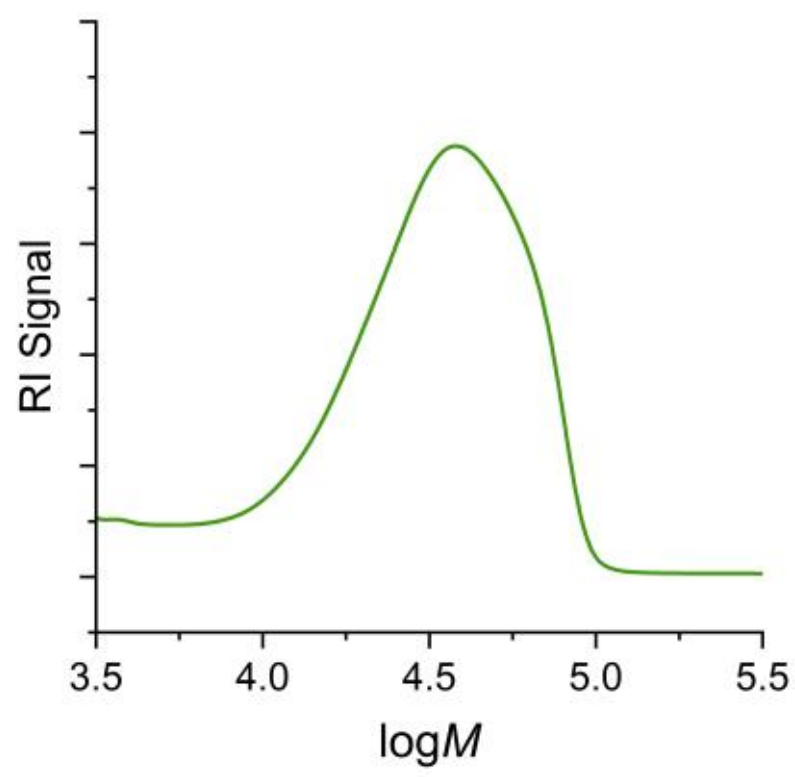

Figure S7. SEC traces of PODP via rROP in toluene ([ODP] = 3M).

Copolymers with ODC

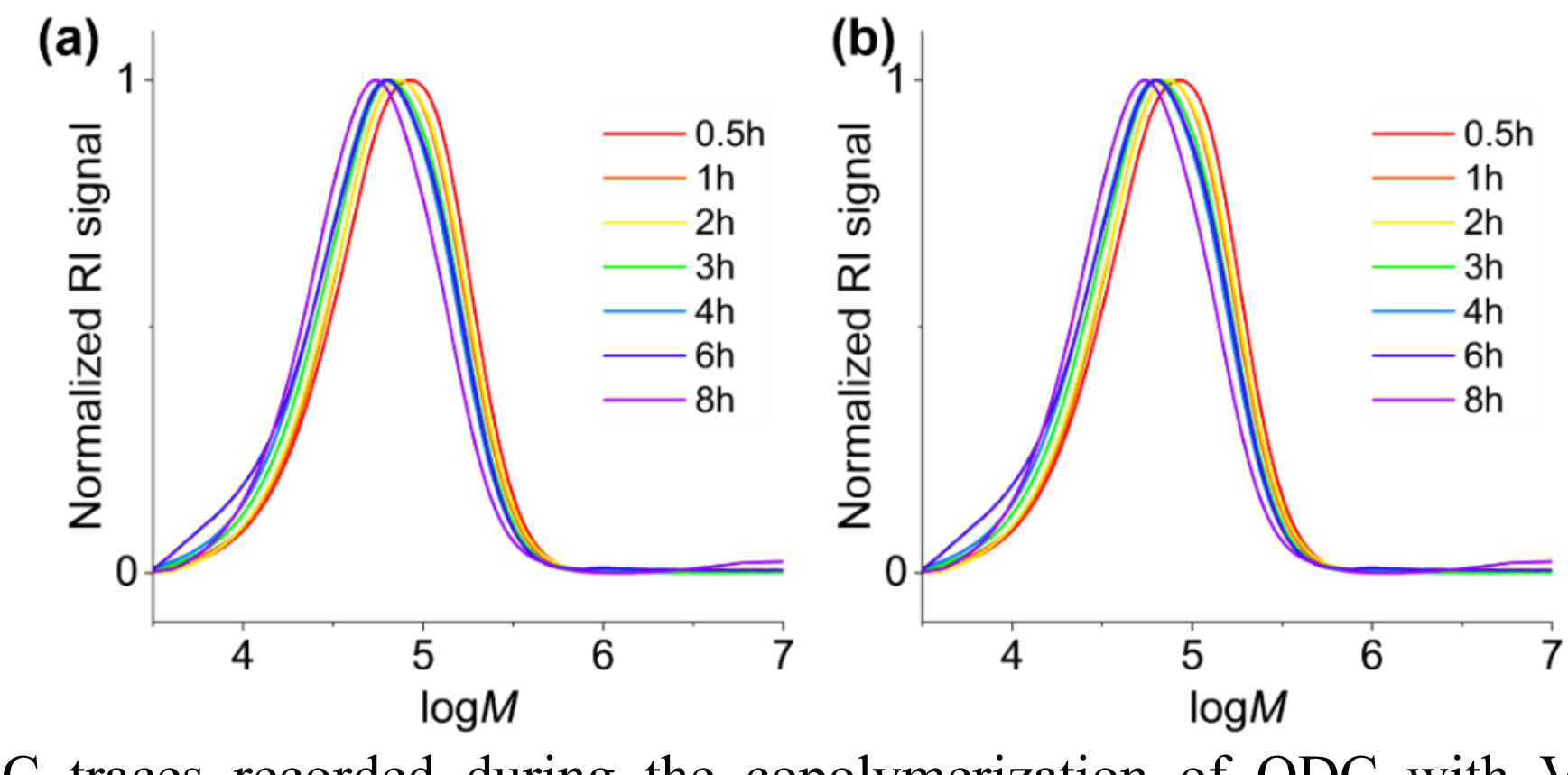

Figure S8. SEC traces recorded during the copolymerization of ODC with VAc, with initial comonomer feed $[VAc]_0$:$[ODC]_0$ = (a) 95:5, (b) 85:15, in bulk at T = 70 °C and 0.5 mol% AIBN.

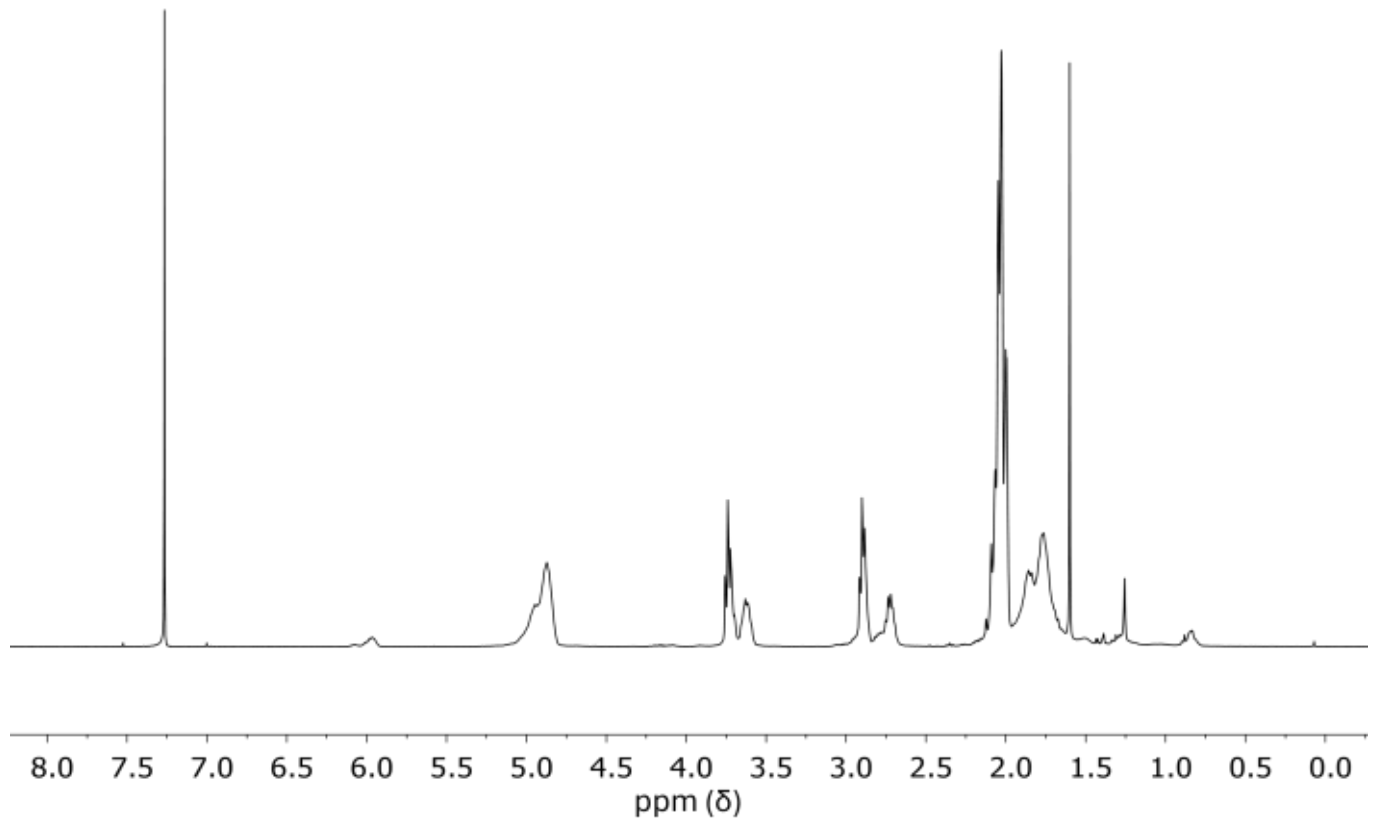

Figure S9. ^{1}H NMR of P(VAc-*co*-ODC) copolymer obtained via FRP of $[VAc]_0:[ODC]_0 = 95:5$.

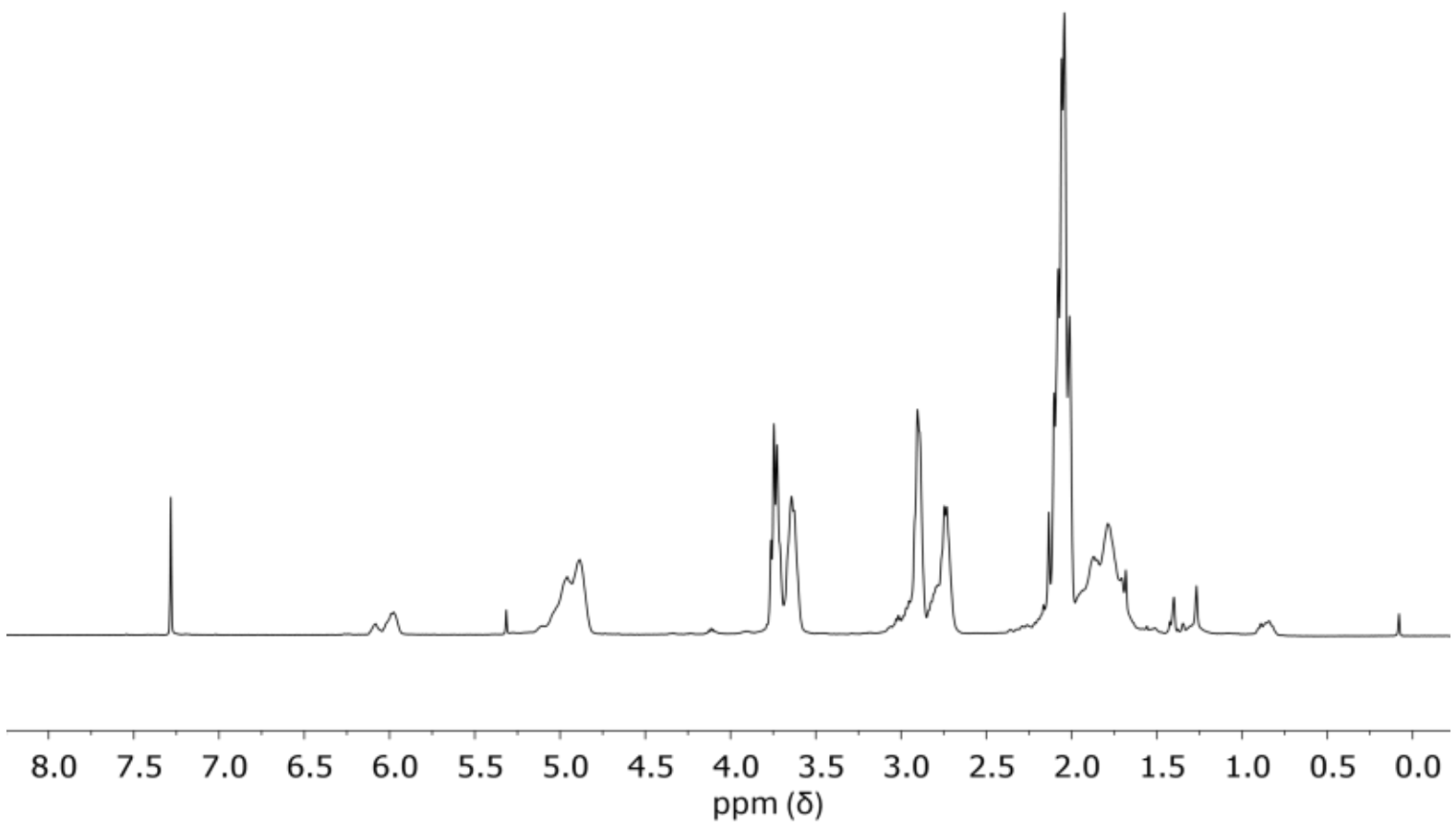

Figure S10. ^{1}H NMR of P(VAc-*co*-ODC) copolymer obtained via FRP of $[VAc]_0:[ODC]_0 = 85:15$.

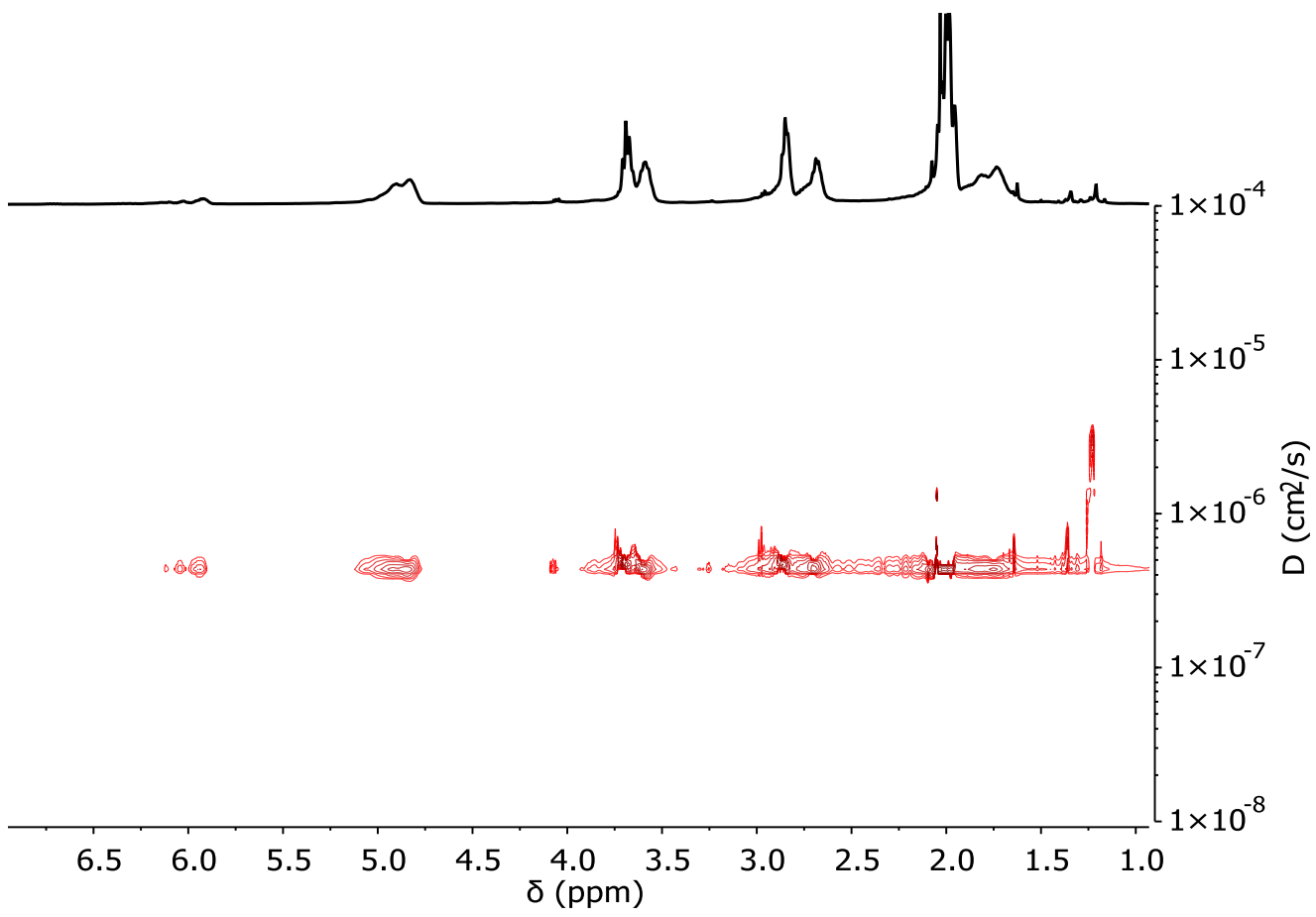

Figure S11. DOSY NMR of purified P(VAc-*co*-ODC) copolymer obtained from the copolymerization of $[VAc]_0:[ODC]_0 = 85:15$ in bulk at $T = 70$ °C.

Synthesis of DEGVE

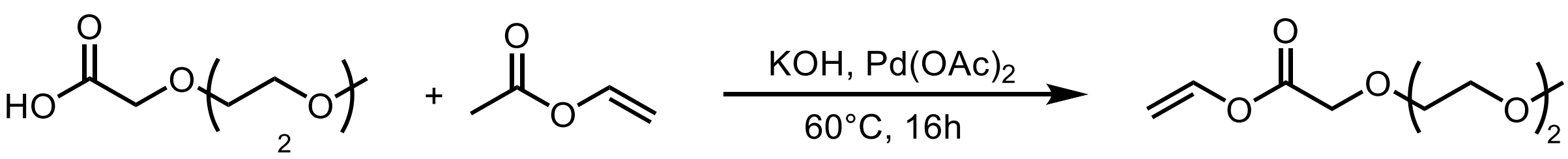

A solution containing [2-(2-methoxyethoxy)ethoxy]acetic acid (10 mL, 11.61 g, 65.16 mmol, 1.0 equiv), KOH (362 mg, 6.45 mmol, 0.10 equiv), $Pd(OAc)_2$ (725 mg, 3.23 mmol, 0.05 equiv), and vinyl acetate (60 mL, 55.58 g, 0.65 mol, 10.0 equiv) was stirred at 60 °C for 16 h in a round-bottom flask.

The reaction mixture was filtered through a Celite and washed thoroughly with diethyl ether. The solvent and residual VAc were removed under reduced pressure. The crude product was purified by column chromatography on silica gel (petroleum ether : EtOAc, 7:3 v/v), followed by vacuum distillation (~1.1 mbar, 130 °C). The product was obtained as a colorless liquid (6.515 g, 50% yield). **^{1}H-NMR** (400 MHz, $CDCl_3$): δ 7.28 (dd, J = 13.9, 6.3 Hz), 4.91 (dd, J = 13.9, 1.8 Hz, 1H), 4.61 (dd, J = 6.3, 1.8 Hz, 1H), 4.23 (s, 2H), 3.78 – 3.50 (m, 8H), 3.35 (s, 3H). **^{13}C-NMR** (101 MHz, $CDCl_3$): δ 167.69, 140.53, 98.49, 68.24, 58.99.

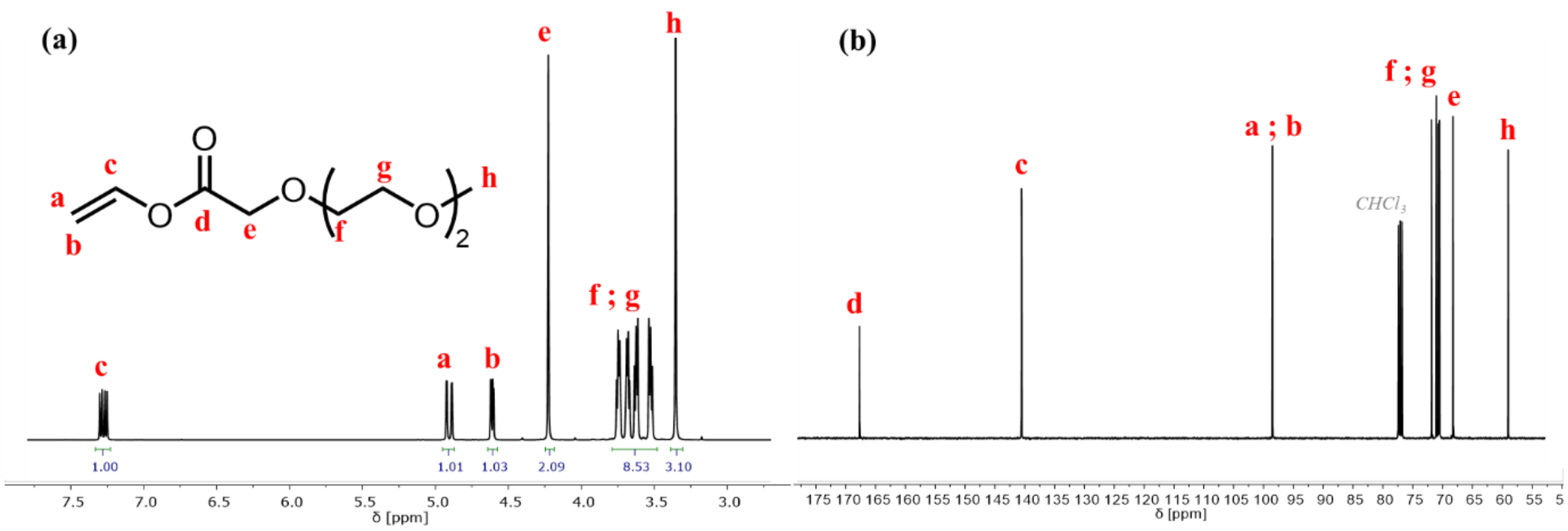

Figure S12. (a) ^{1}H NMR and (b) ^{13}C NMR spectra in $CDCl_3$ of DEGVE.

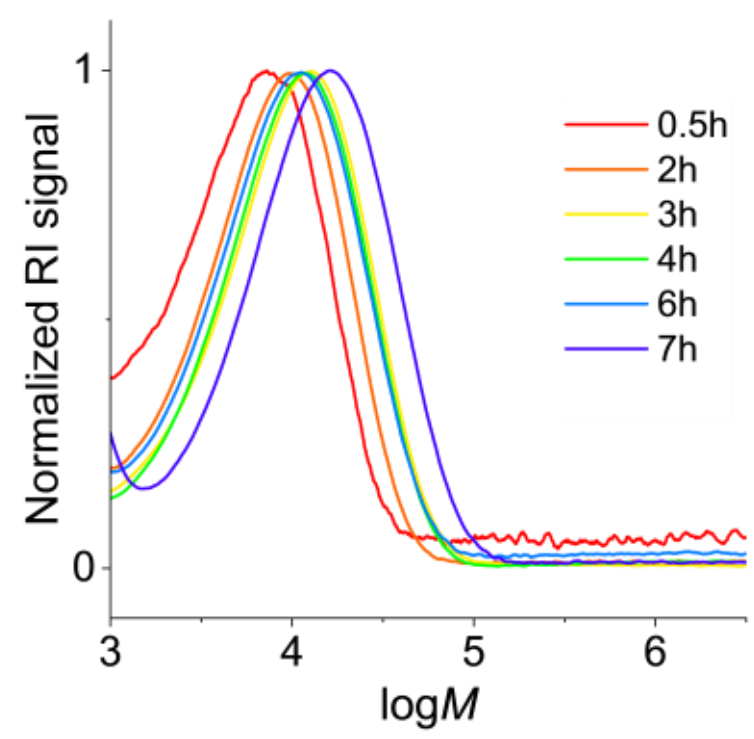

Figure S13. SEC traces recorded during the copolymerization of ODC with DEGVE, with initial comonomer feed $[DEGVE]_0:[ODC]_0$ = 85:15, in bulk at T = 70 °C and 2 mol% AIBN.

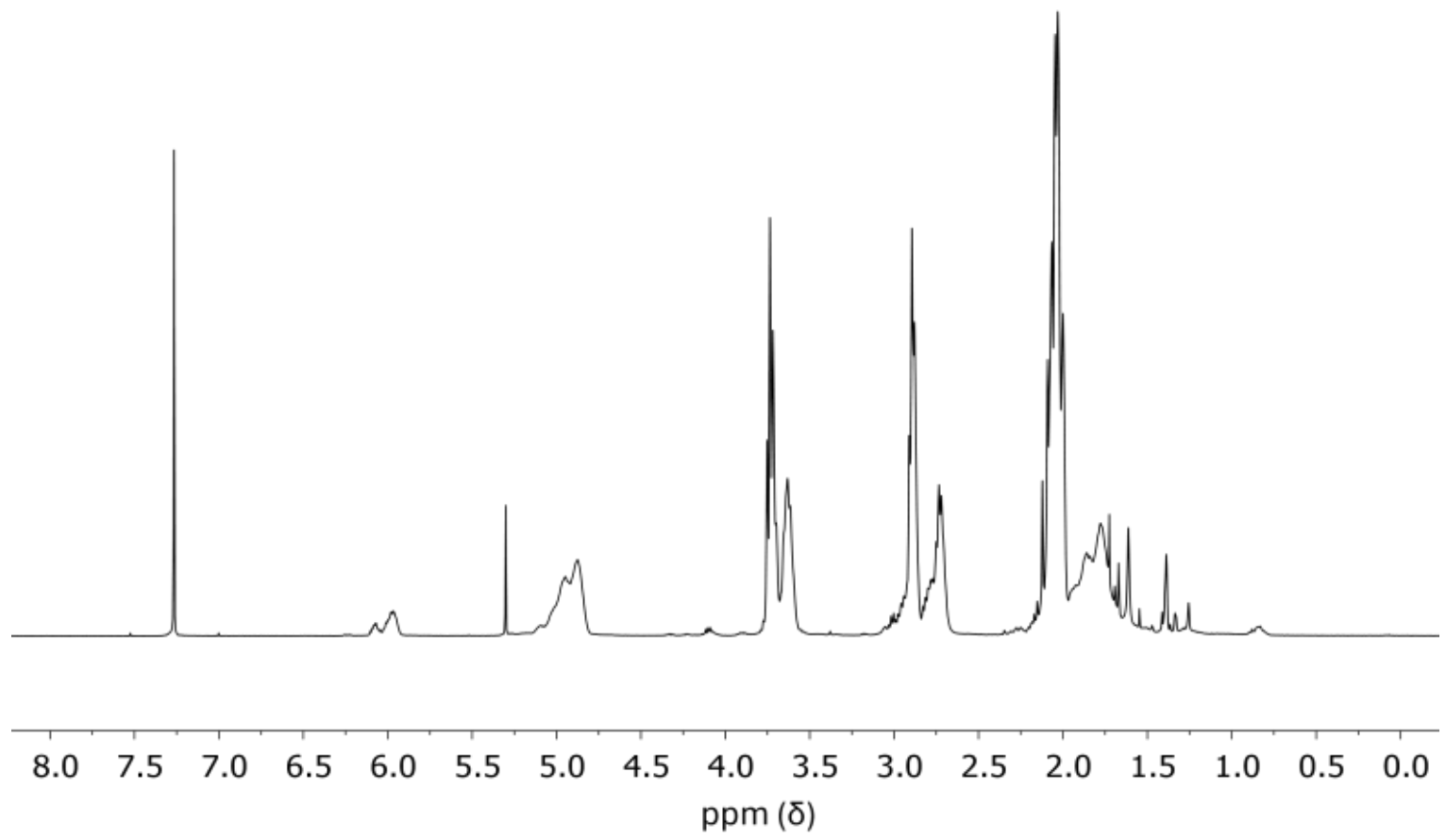

Figure S14. ^{1}H NMR of P(DEGVE-*co*-ODC) copolymer obtained via FRP of $[DEGVE]_0$:$[ODC]_0$ = 85:15.

Synthesis of 2-(methyl propionate)-O-ethyl xanthate

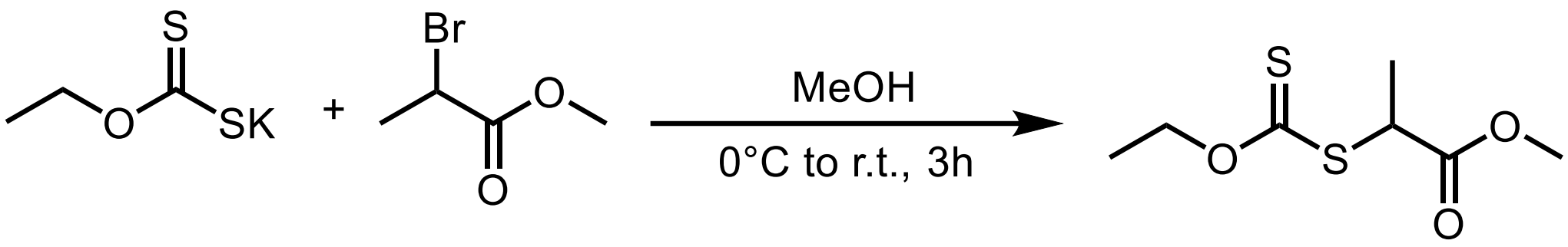

In a 100 mL round-bottom flask, 5.001 g of methyl 2-bromopropanoate (30 mmol, 1 eq.) were dissolved in 50 mL of methanol (MeOH). After cooling the solution in an ice bath, 5.324 g of potassium *O*-ethyl dithiocarbonate (33 mmol, 1.1 eq.) were added dropwise over 45 minutes, resulting in the formation of a yellow solution. The reaction mixture was then removed from the ice bath and stirred at room temperature for 3 hours. The formation of a white solid can be observed.

The resulting solid was separated by Büchner filtration, and the solvent was removed under reduced pressure using a rotary evaporator. The residue was dissolved in 75 mL of dichloromethane and washed with water (5 × 50 mL) until a clear organic phase was obtained. The organic layer was dried over anhydrous Na_2SO_4, filtered and concentrated under reduced pressure. The desired compound was obtained as an yellow oily (yield: 89%). **^{1}H-NMR** (400 MHz, $CDCl_3$): δ 4.42 (qd, *J* = 7.1, 1.6 Hz, 1H), 3.54 (s, 1H), 1.36 (d, *J* = 7.4 Hz, 1H), 1.20 (t, *J* = 7.1 Hz, 1H).**^{13}C-NMR** (101 MHz, $CDCl_3$): 212.04, 171.93, 70.30, 52.78, 47.03, 16.90, 13.68.

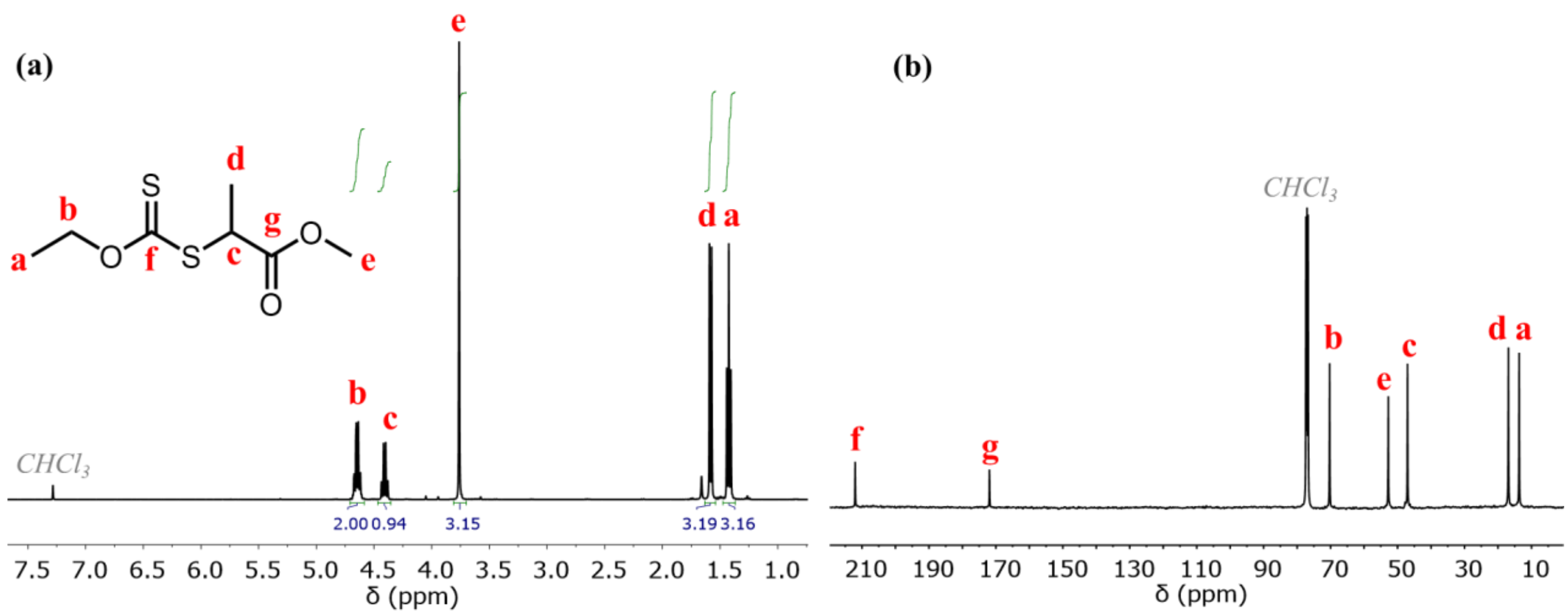

Figure S15. (a) ^{1}H NMR and (b) ^{13}C NMR spectra in $CDCl_3$ of the xanthate chain transfer agent (CTA).

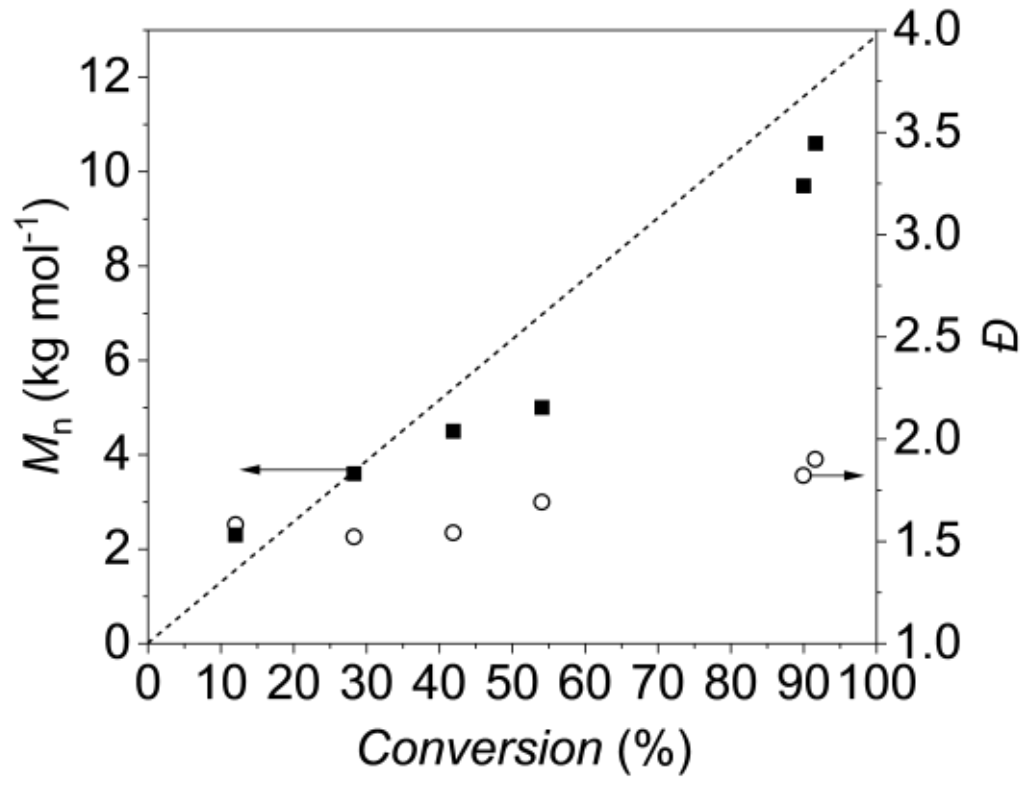

Figure S16. Evolution of molar mass and dispersity with total monomer conversion for the copolymerization of ODC with VAc via PI-RAFT/MADIX, with initial comonomer feed $[VAc]_0$:$[ODC]_0$ = 85:15, and ($[VAc]_0$:$[ODC]_0$):[CTA] = 150, in bulk under blue light irradiation (λ_{max} = 427 nm).

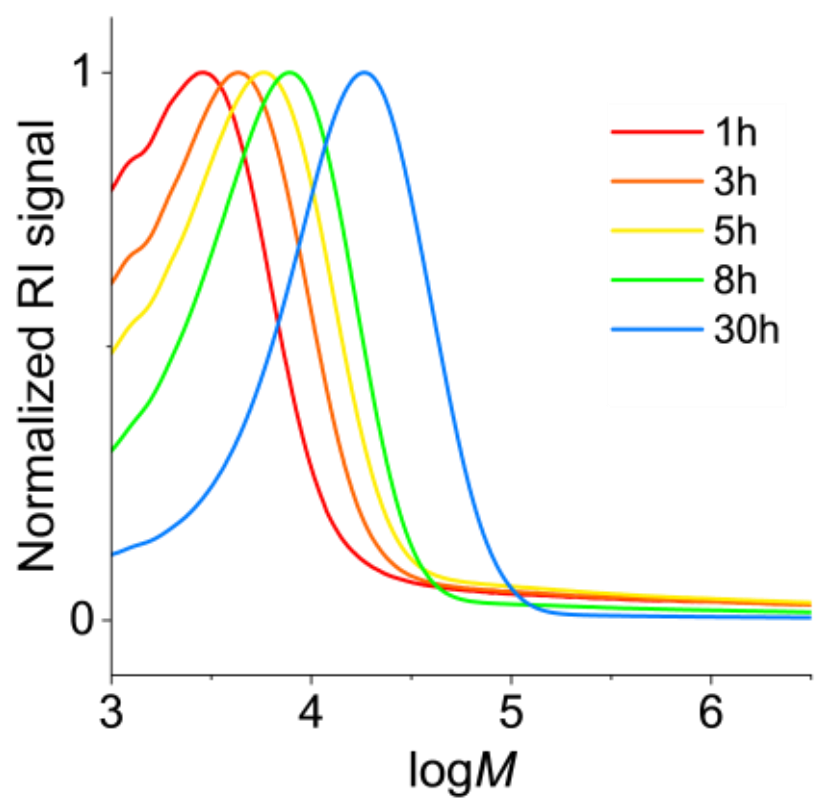

Figure S17. SEC traces recorded during the copolymerization of ODC with VAc, with initial comonomer feed $[VAc]_0$:$[ODC]_0$ = 85:15, and ($[VAc]_0$:$[ODC]_0$):[CTA] = 150, in bulk under blue light irradiation (λ_{max} = 427 nm).

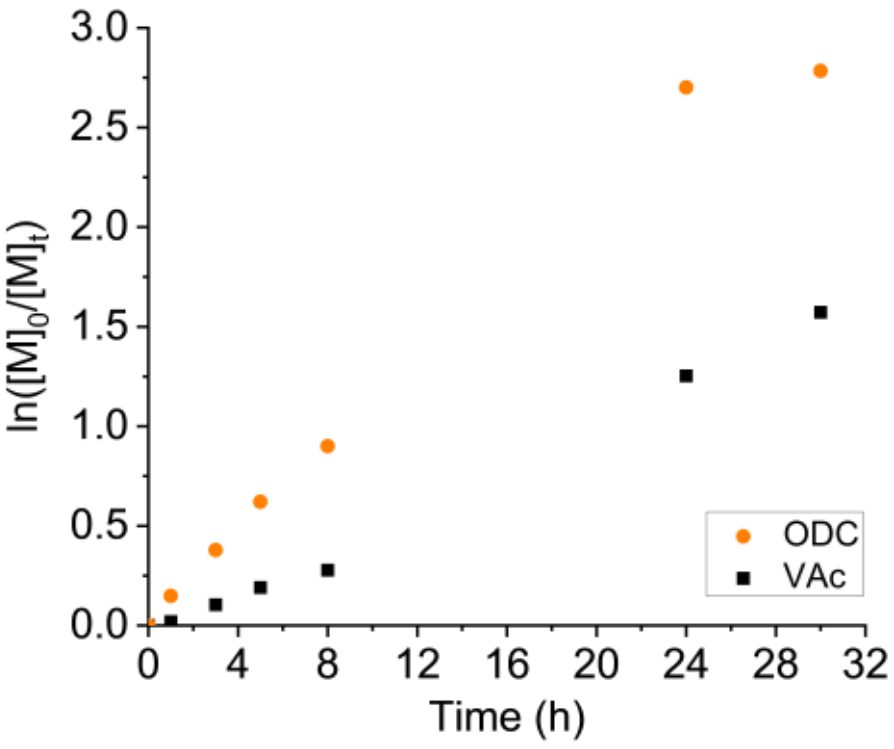

Figure S18. Kinetics of copolymerization of ODC with VAc via PI-RAFT/MADIX polymerization, with initial comonomer feed $[VAc]_0:[ODC]_0$ = 85:15, and $([VAc]_0:[ODC]_0):[CTA]$ = 150, in bulk under blue light irradiation (λ_{max} = 427 nm).

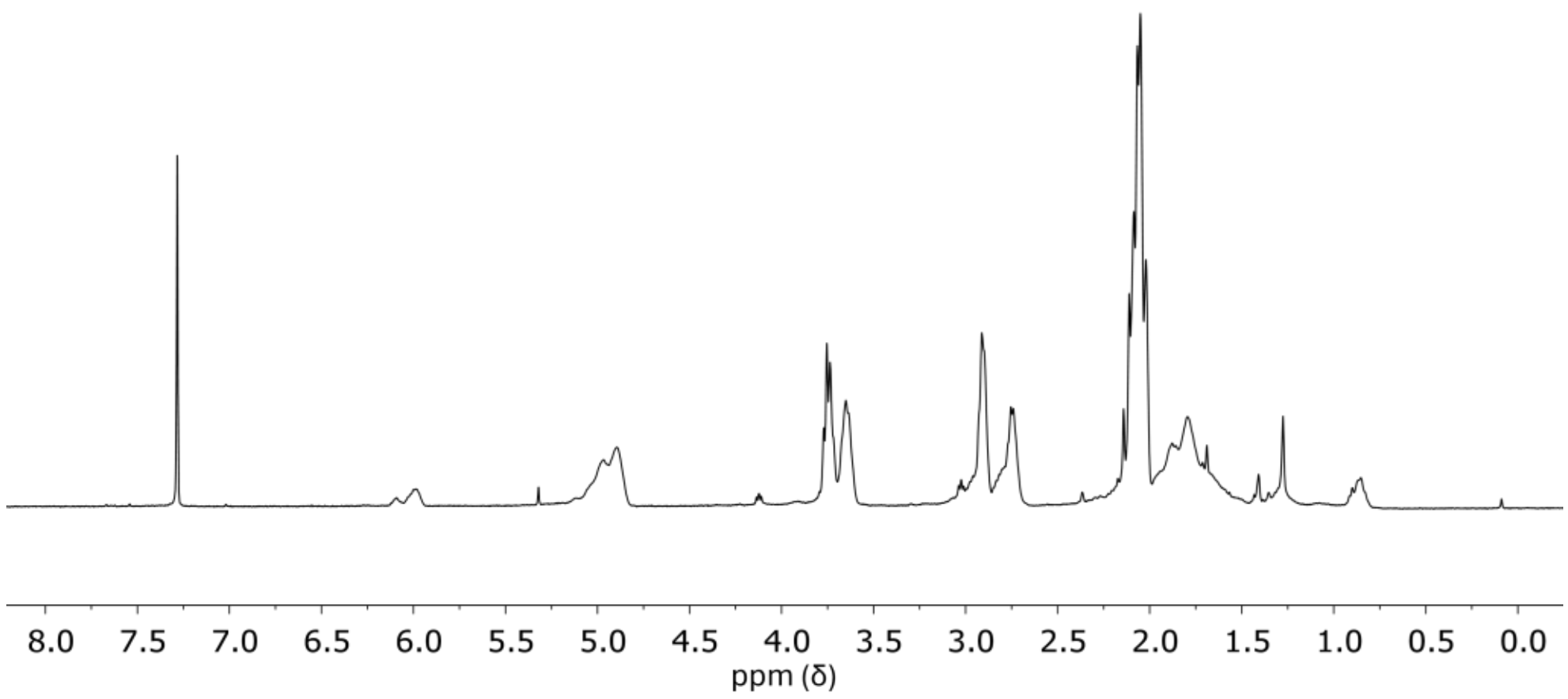

Figure S19. ^{1}H NMR of P(VAc-*co*-ODC) copolymer obtained via PI-RAFT/MADIX polymerization of $[VAc]_0:[ODC]_0$ = 85:15.

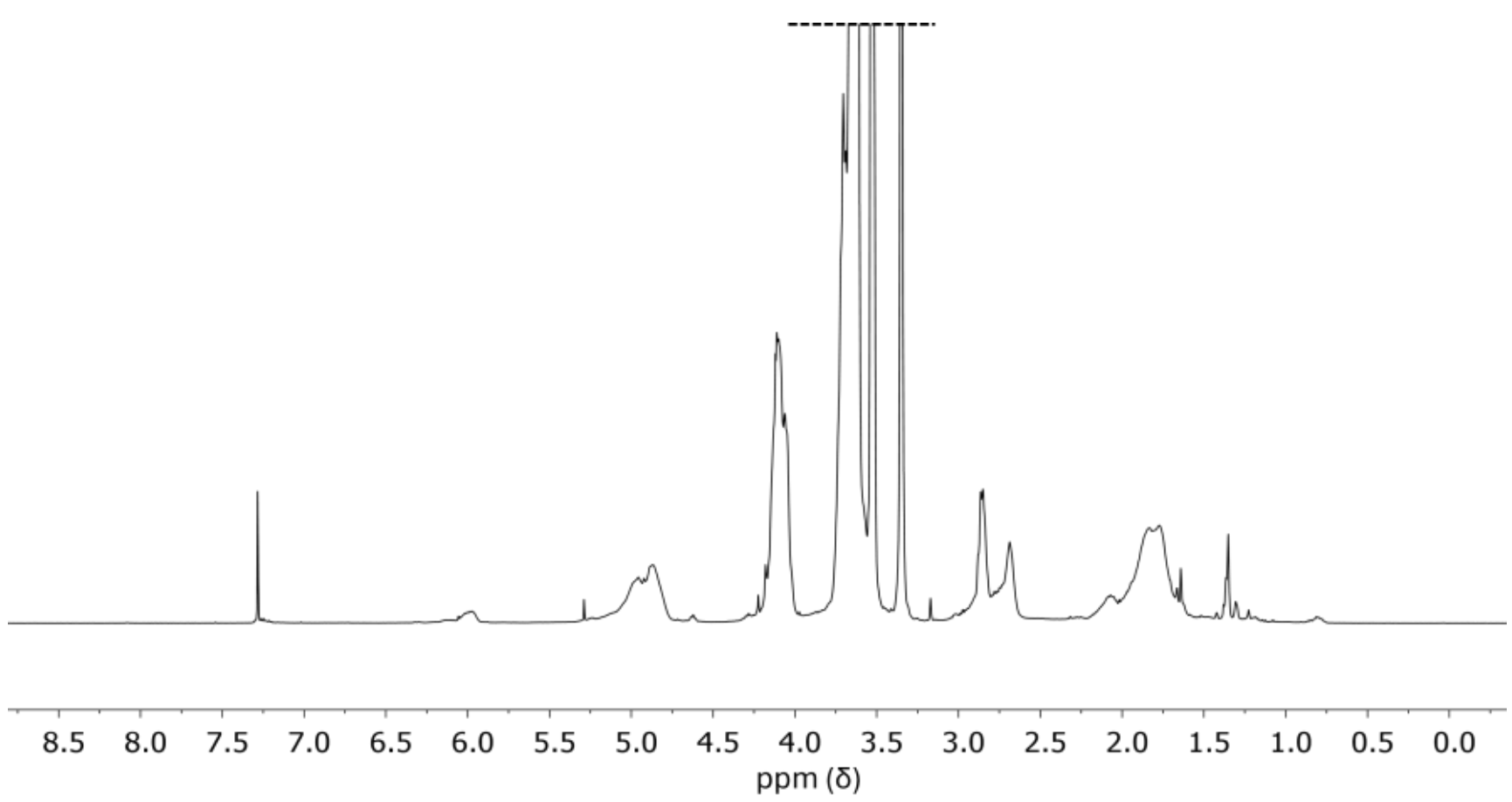

Figure S20. ^{1}H NMR of P(DEGVE-*co*-ODC) copolymer obtained via PI-RAFT/MADIX polymerization of $[DEGVE]_0:[ODC]_0$ = 85:15.

Copolymers with ODP

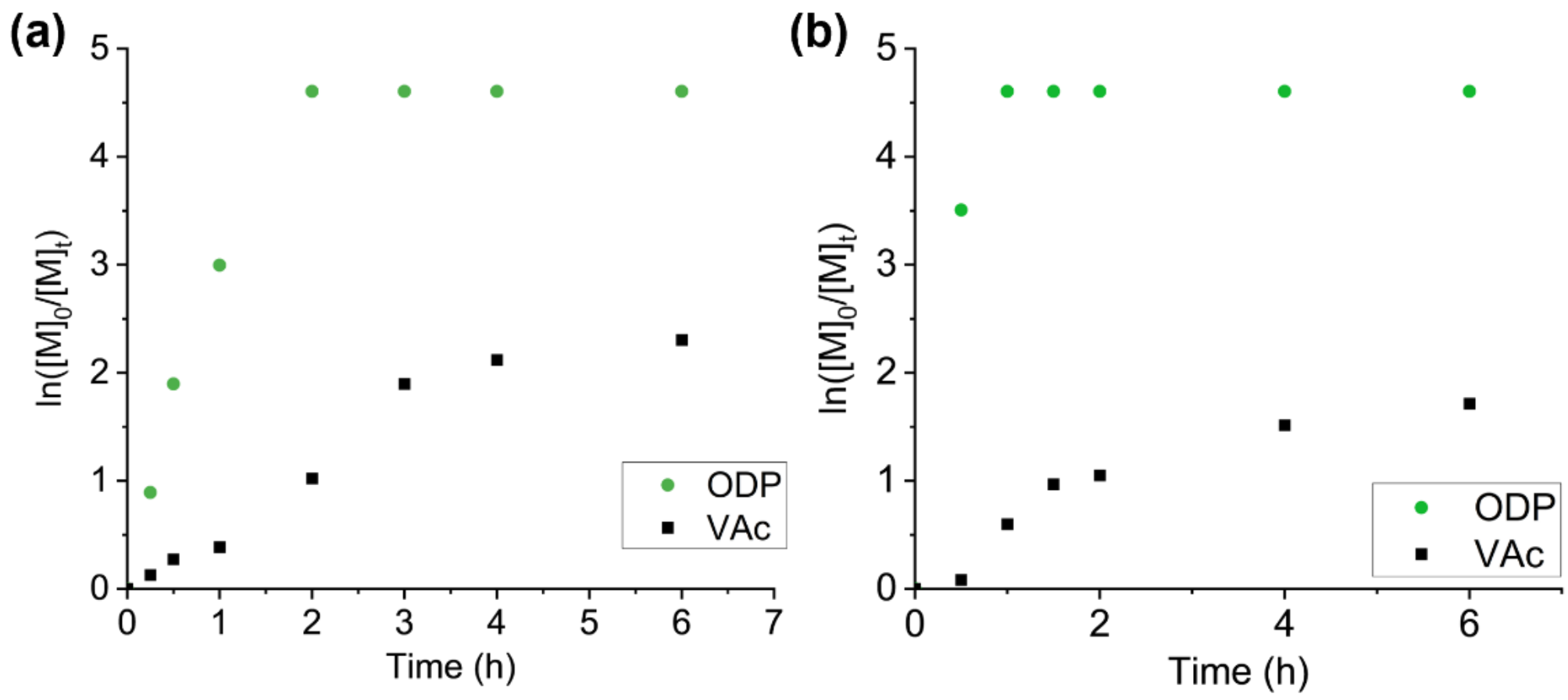

Figure S21. Kinetics of copolymerization of ODP with VAc, with initial comonomer feed $[VAc]_0$:$[ODP]_0$ = (a) 95:5, (b) 85:15, in bulk at T = 70 °C and 2 mol% AIBN.

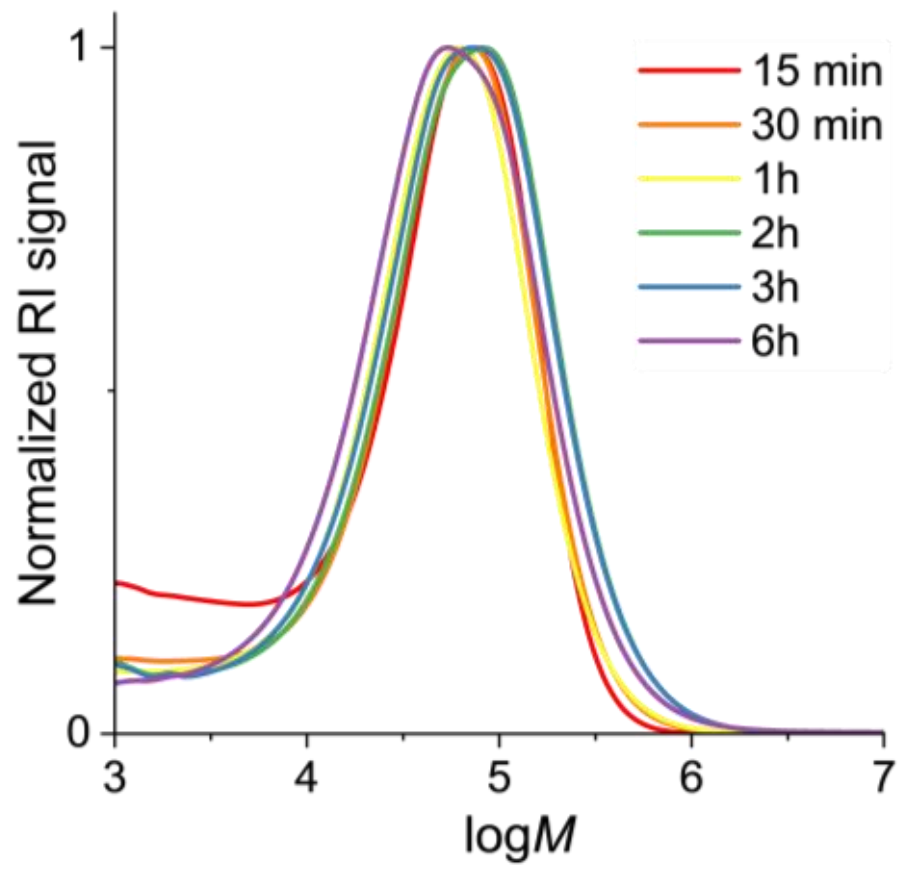

Figure S22. SEC traces recorded during the copolymerization of ODP with VAc, with initial comonomer feed $[VAc]_0$:$[ODP]_0$ = 95:5 in bulk at 70 °C.

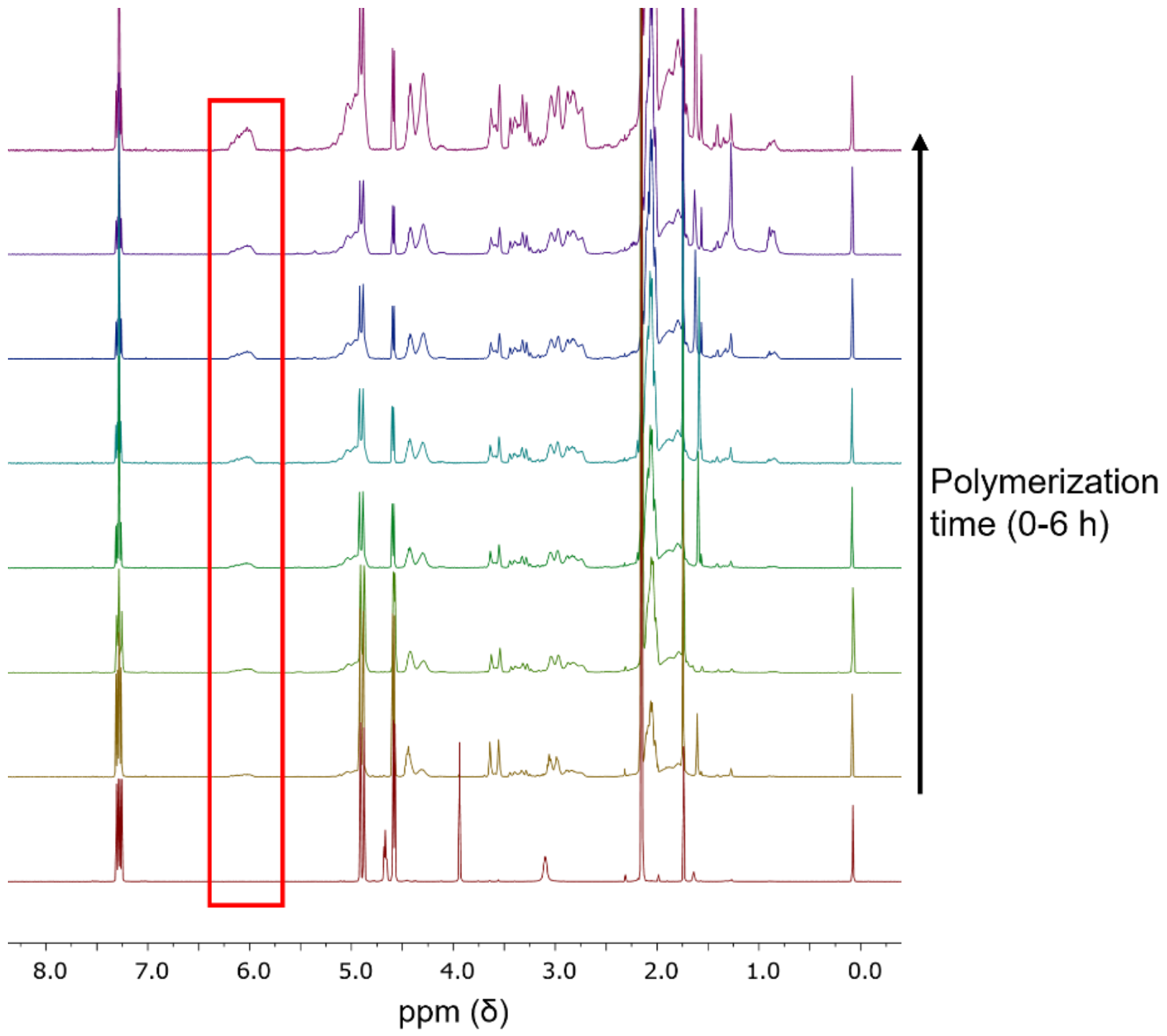

Figure S23. ^{1}H NMR spectra recorded during the copolymerization of ODP with VAc, with initial comonomer feed $[VAc]_0$:$[ODP]_0$ = 85:15, in bulk at T = 70 °C and 2 mol% AIBN. The red frame highlights the signal attributed to the methine proton of a VAc unit linked to a S atom, -CH_2-C***H***($OOCCH_3$)-S-, whose intensity increases even after complete consumption of ODP (~2 h after the FRP start).

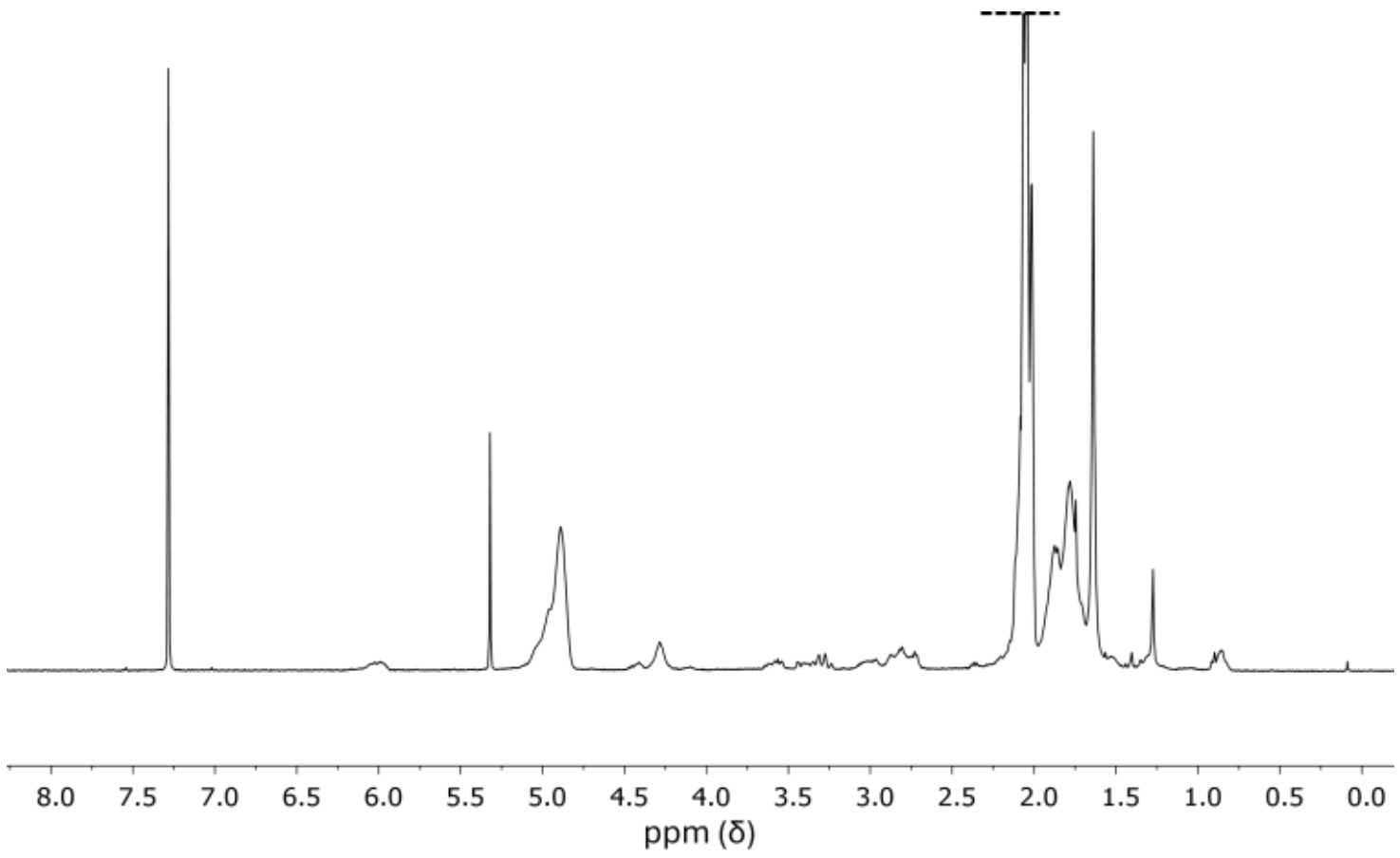

Figure S24. ^{1}H NMR of P(VAc-*co*-ODP) copolymer obtained via FRP of $[VAc]_0$:$[ODP]_0$ = 95:5.

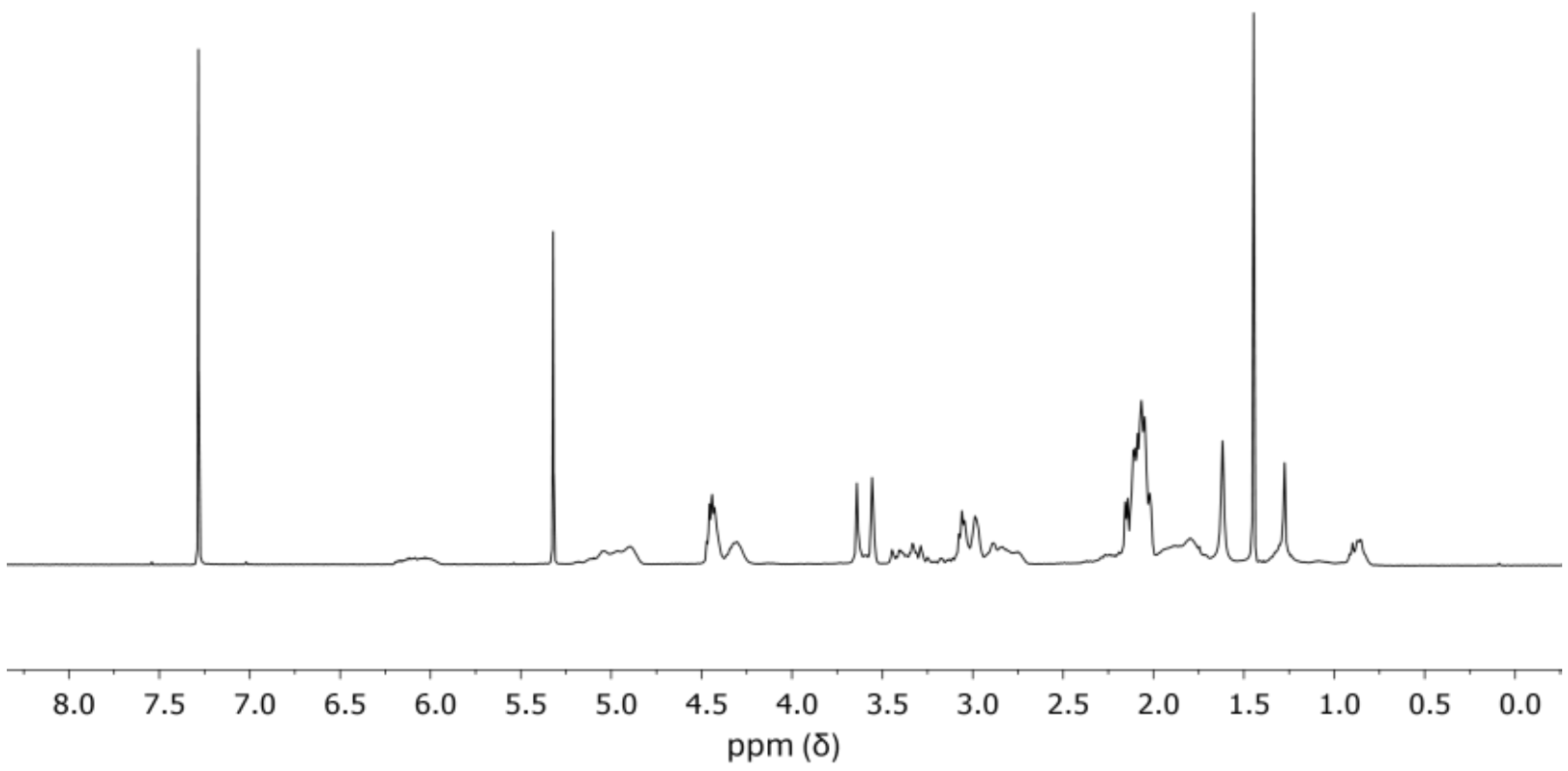

Figure S25. ^{1}H NMR of P(VAc-co-ODP) copolymer obtained via FRP of $[VAc]_0$:$[ODP]_0$ = 85:15.

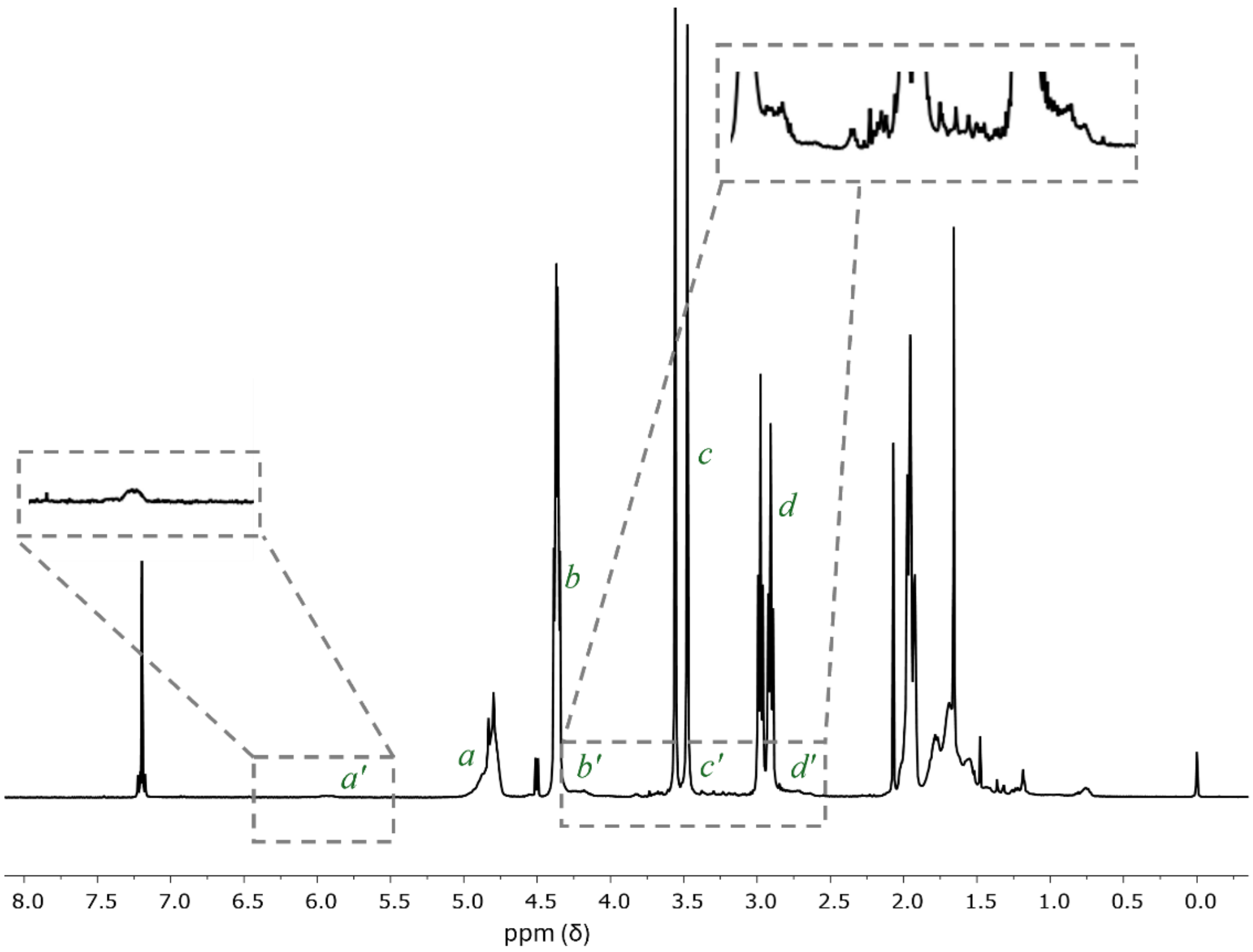

Figure S26. ^{1}H NMR spectrum recorded during the FRP of VAc in the presence of PODP (t = 5 h), with $[VAc]_0:[ODP]_0$ = 85:15. Dashed frame highlight the signals attributed to ODP-VAc and VAc-ODP dyads indicating that chain transfer to S-S is occurring during the polymerization of VAc.

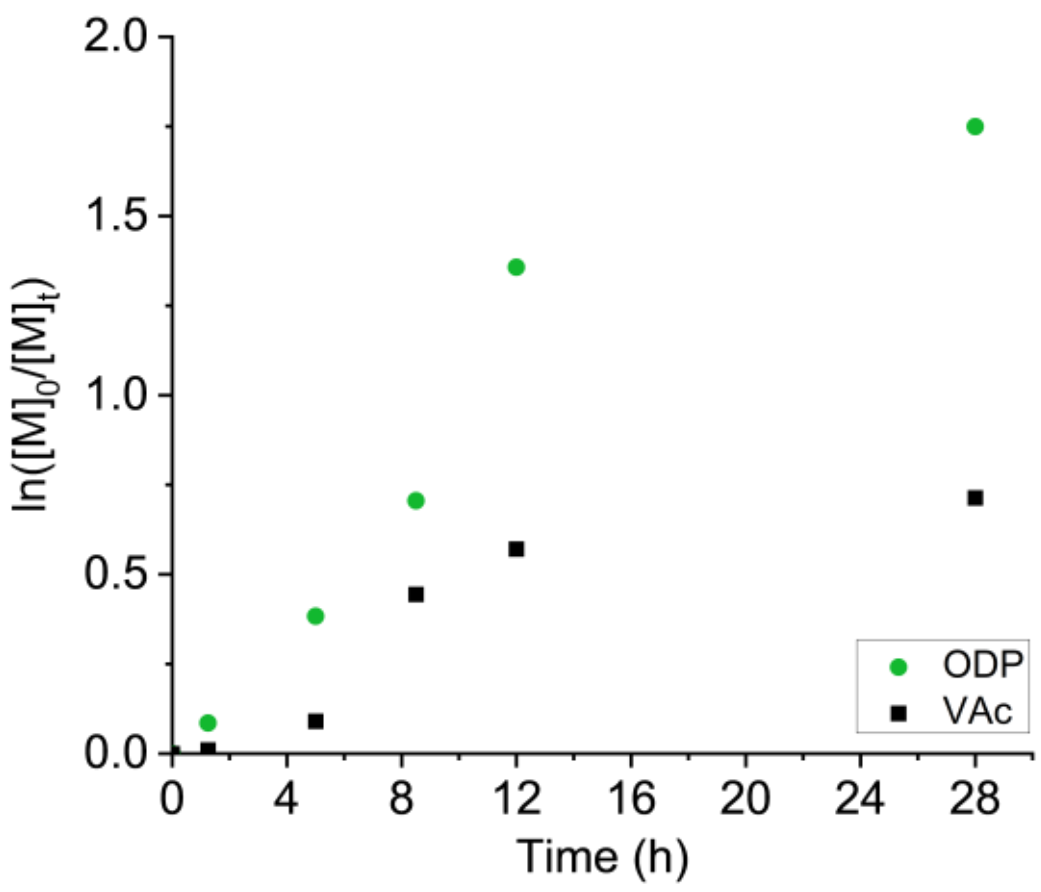

Figure S27. Kinetics of copolymerization of ODP with VAc via PI-RAFT/MADIX, with initial comonomer feed $[VAc]_0:[ODP]_0$ = 95:5, and $([VAc]_0:[ODP]_0):[CTA]$ = 150, in bulk under blue light irradiation (λ_{max} = 427 nm).

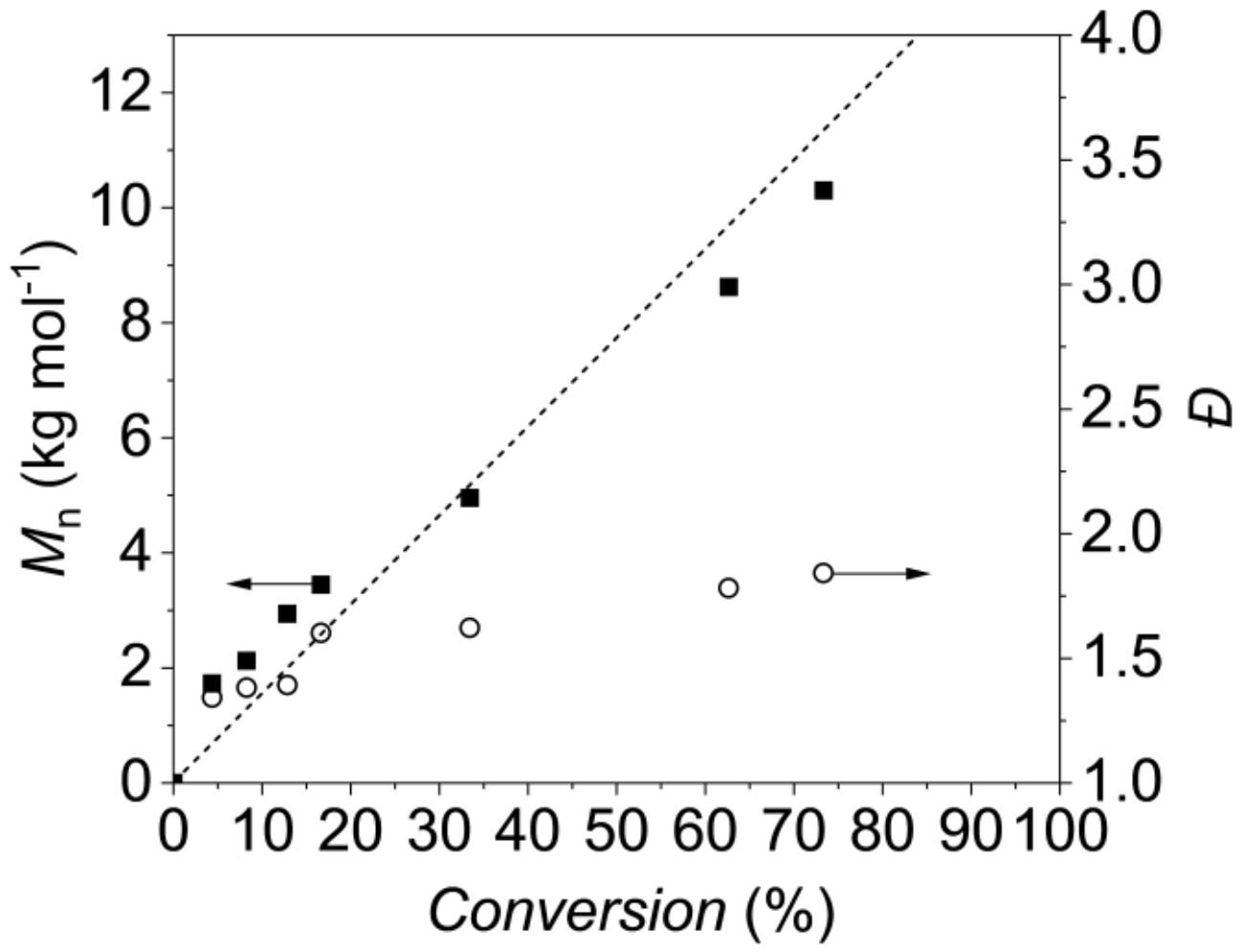

Figure S28. Evolution of molar mass and dispersity with total monomer conversion for the copolymerization of ODP with VAc via PI-RAFT/MADIX, with initial comonomer feed $[VAc]_0$:$[ODP]_0$ = 85:15, and ($[VAc]_0$:$[ODP]_0$):[CTA] = 150, in bulk under blue light irradiation (λ_{max} = 427 nm).

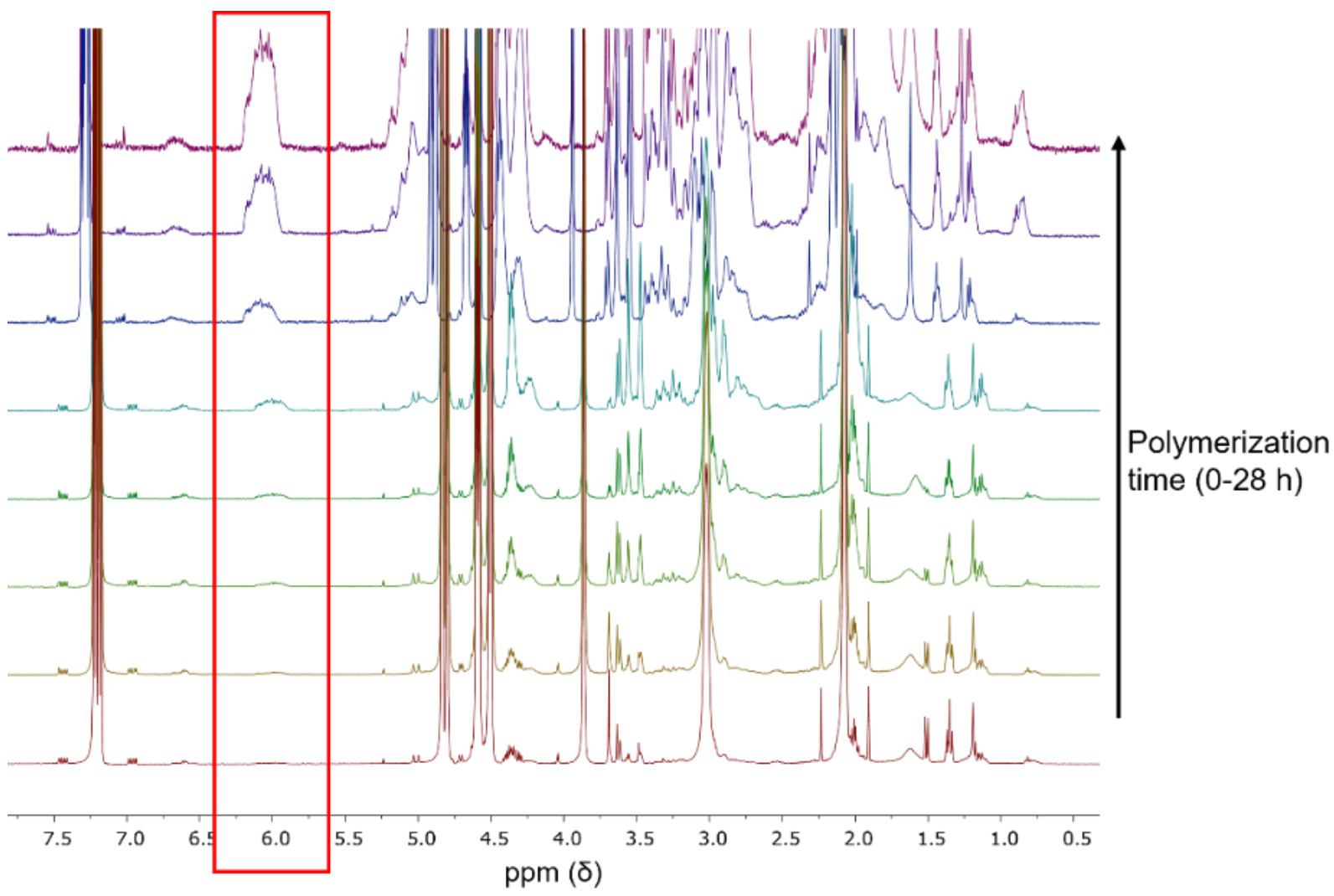

Figure S29. ^{1}H NMR spectra recorded during the copolymerization of ODP with VAc via PI-RAFT/MADIX, with initial comonomer feed $[VAc]_0$:$[ODP]_0$ = 85:15. The red frame highlights the signal attributed to the methine proton of a VAc unit linked to a S atom, -CH_2-C***H***($OOCCH_3$)-S-, whose intensity increases with the progressive consumption of ODP.

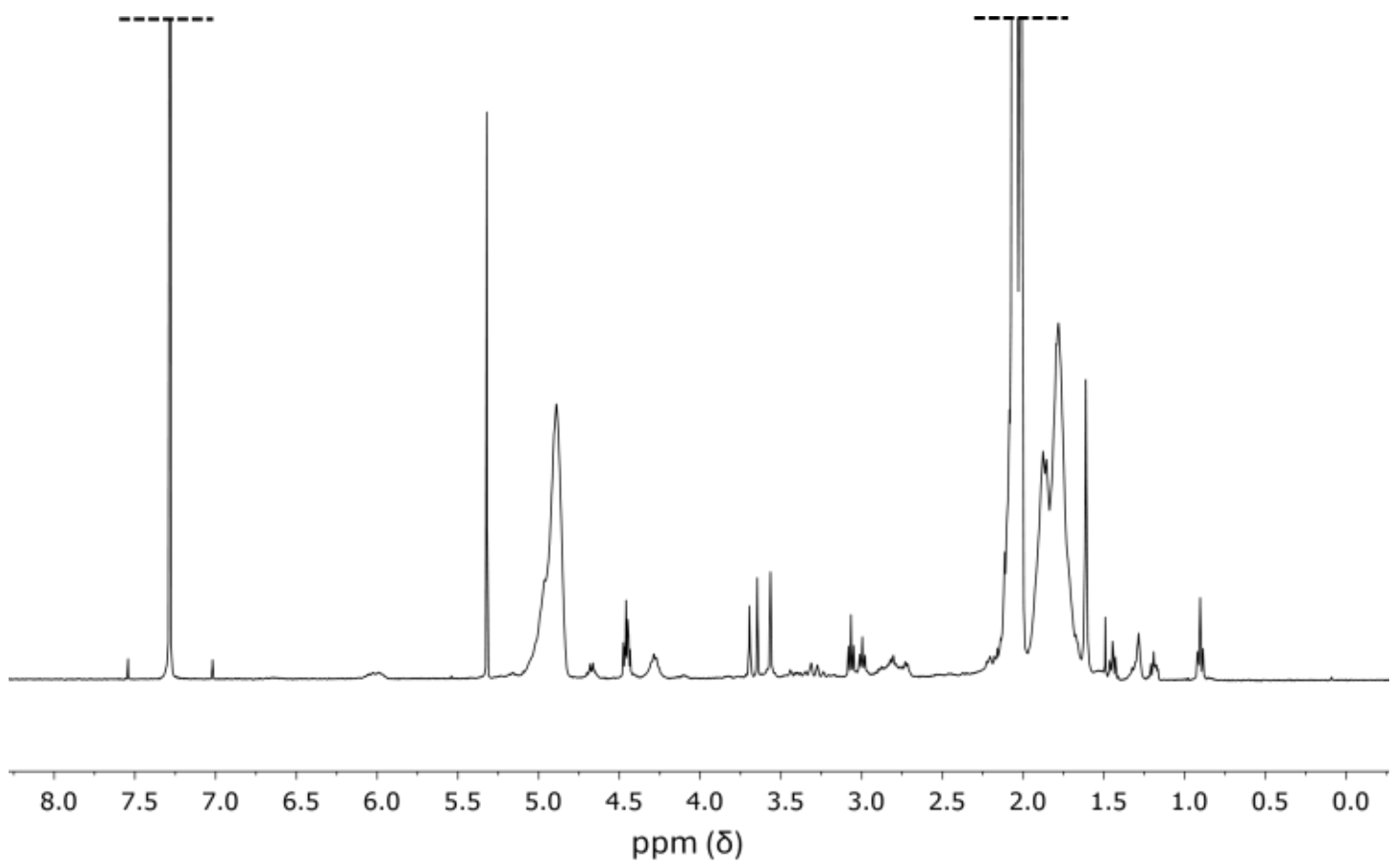

Figure S30. ^{1}H NMR of P(VAc-*co*-ODP) copolymer obtained via PI-RAFT/MADIX polymerization of $[VAc]_0$:$[ODP]_0$ = 95:5.

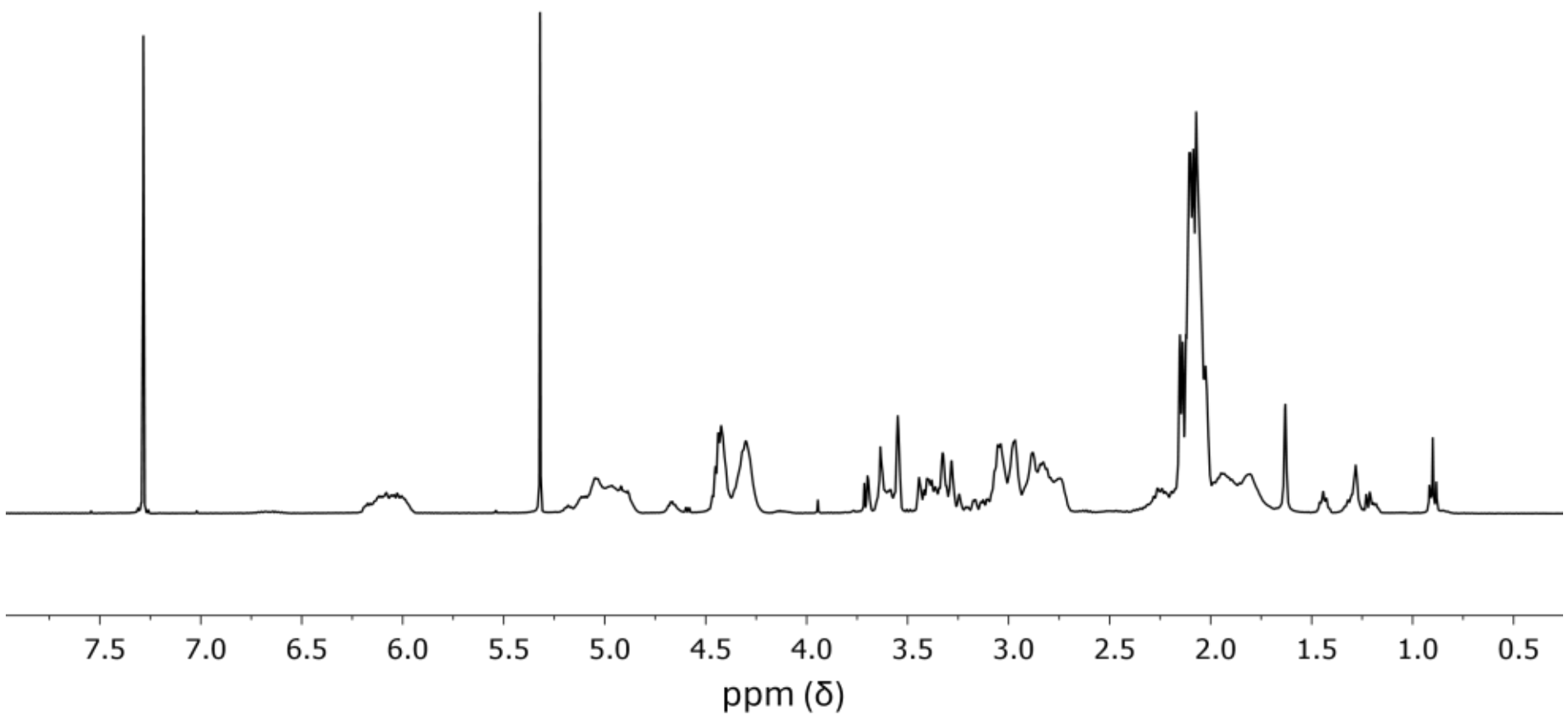

Figure S31. ^{1}H NMR of P(VAc-*co*-ODP) copolymer obtained via PI-RAFT/MADIX polymerization of $[VAc]_0$:$[ODP]_0$ = 85:15.

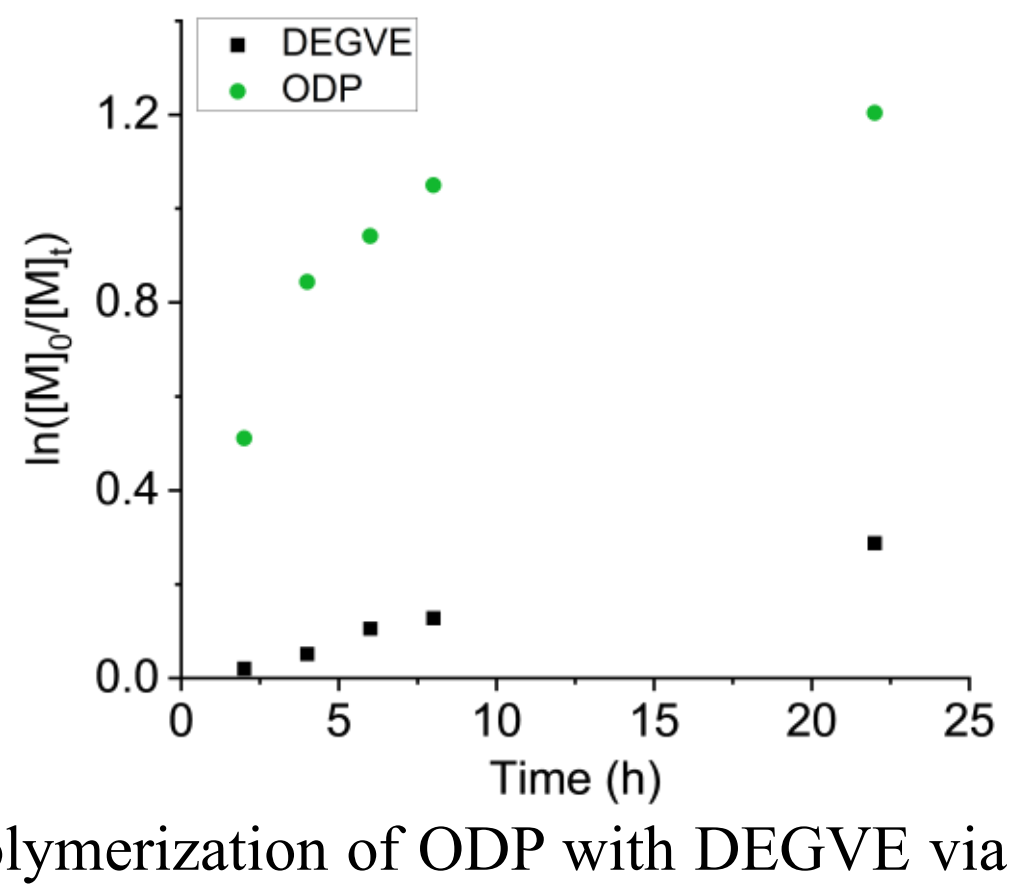

Figure S32. Kinetics of copolymerization of ODP with DEGVE via PI-RAFT/MADIX, with initial comonomer feed $[DEGVE]_0$:$[ODP]_0$ = 85:15, and ($[DEGVE]_0$:$[ODP]_0$):[CTA] = 150, in bulk under blue light irradiation (λ_{max} = 427 nm).

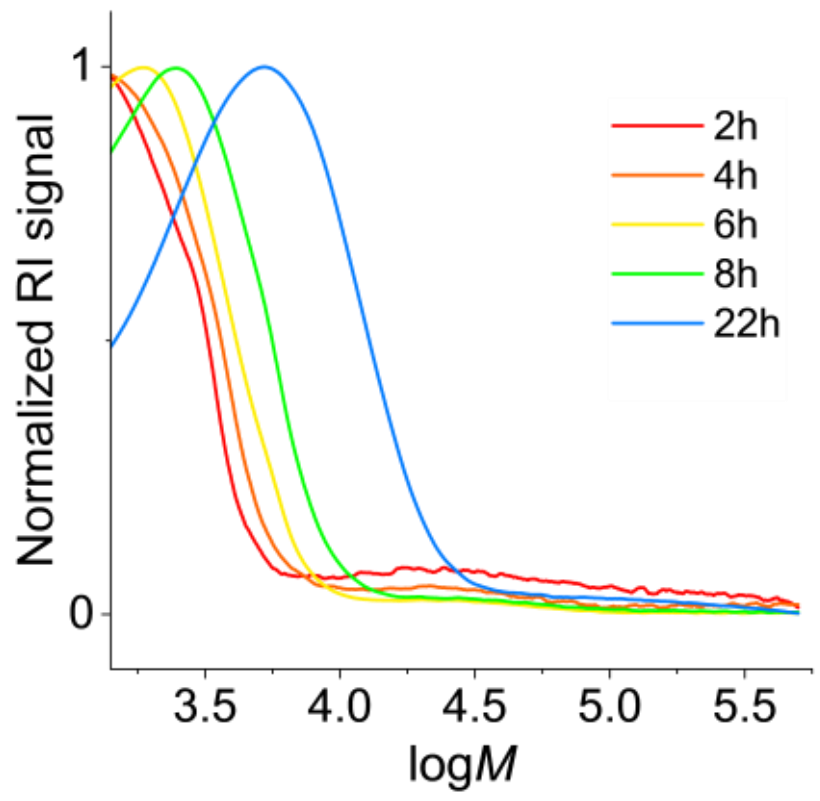

Figure S33. SEC traces recorded during the copolymerization of DEGVE with ODP, with initial comonomer feed $[DEGVE]_0:[ODP]_0$ = 85:15, and $([DEGVE]_0:[ODP]_0):[CTA]$ = 150, in bulk under blue light irradiation (λ_{max} = 427 nm).

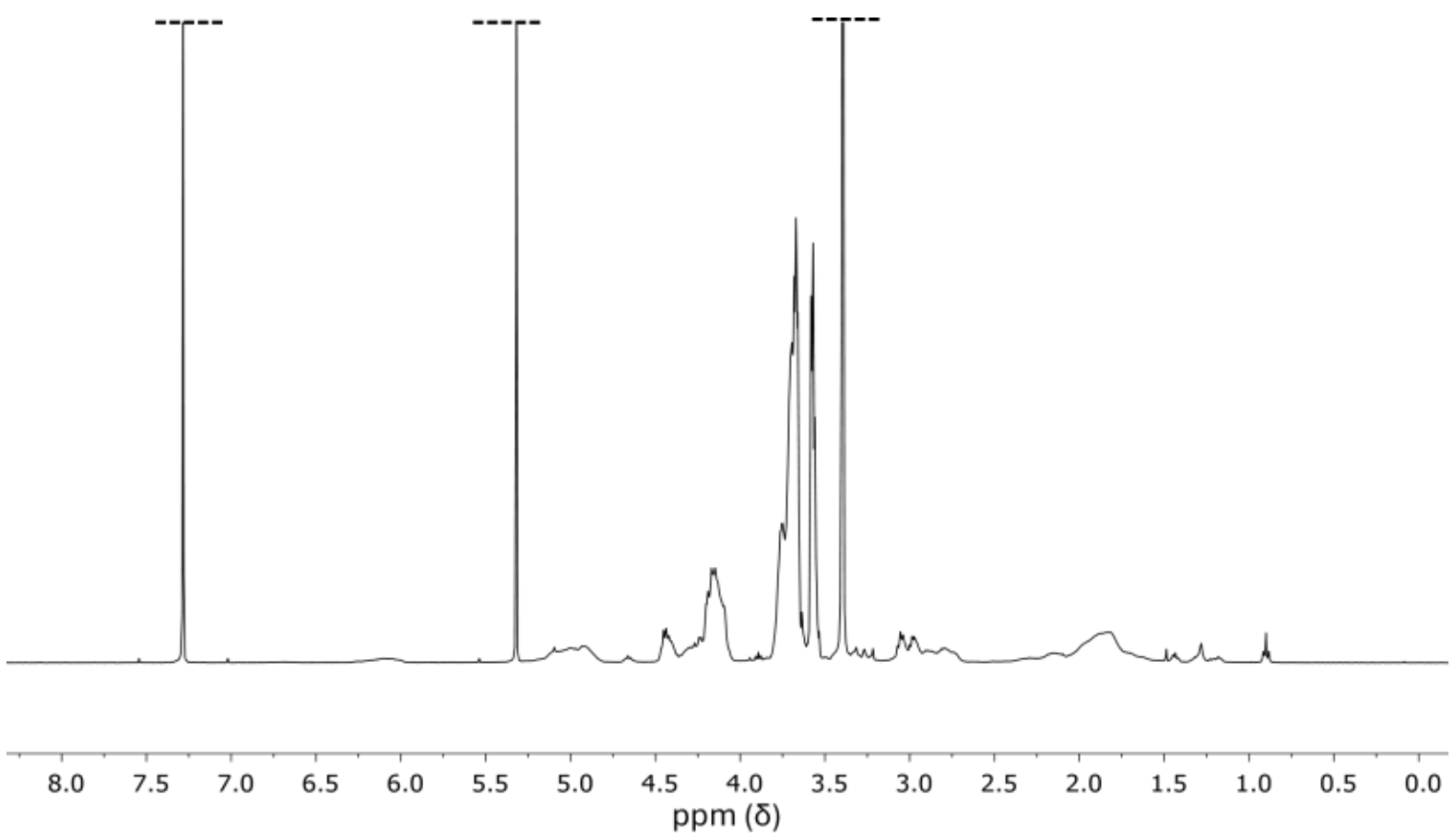

Figure S34. ^{1}H NMR of P(DEGVE-*co*-ODP) copolymer obtained via PI-RAFT/MADIX polymerization of $[DEGVE]_0:[ODP]_0$ = 85:15.

Degradation of Copolymers

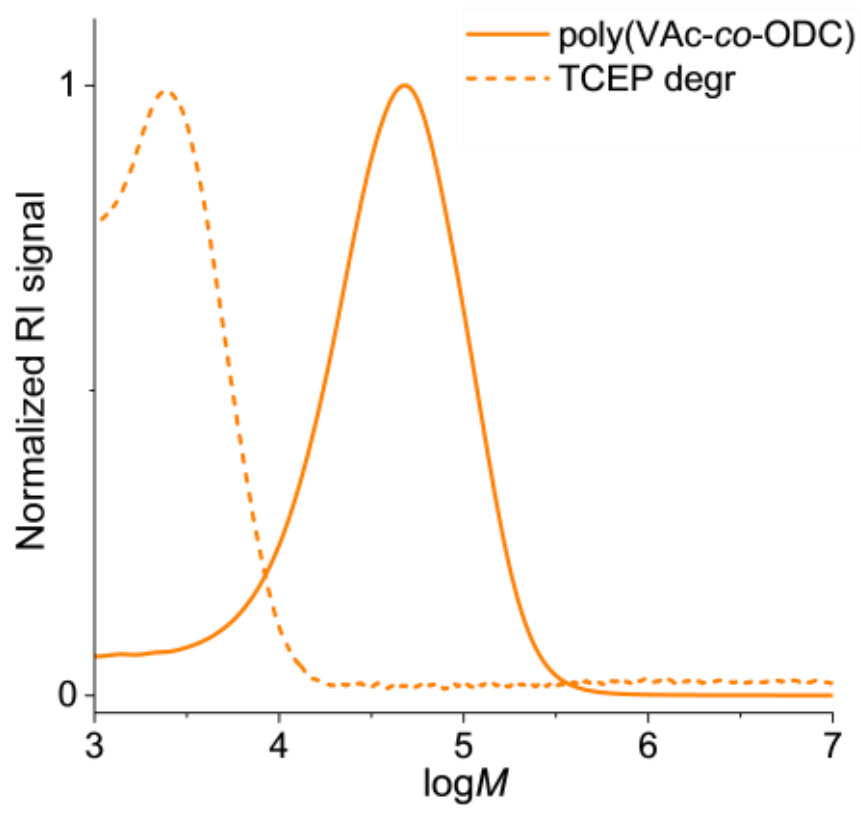

Figure S35. SEC traces recorded before and after degradation with TCEP of the copolymers of ODC with VAc, with initial comonomer feed $[VAc]_0:[ODC]_0$ = 85:15, prepared via FRP in bulk at T = 70 °C and 0.5 mol% AIBN.

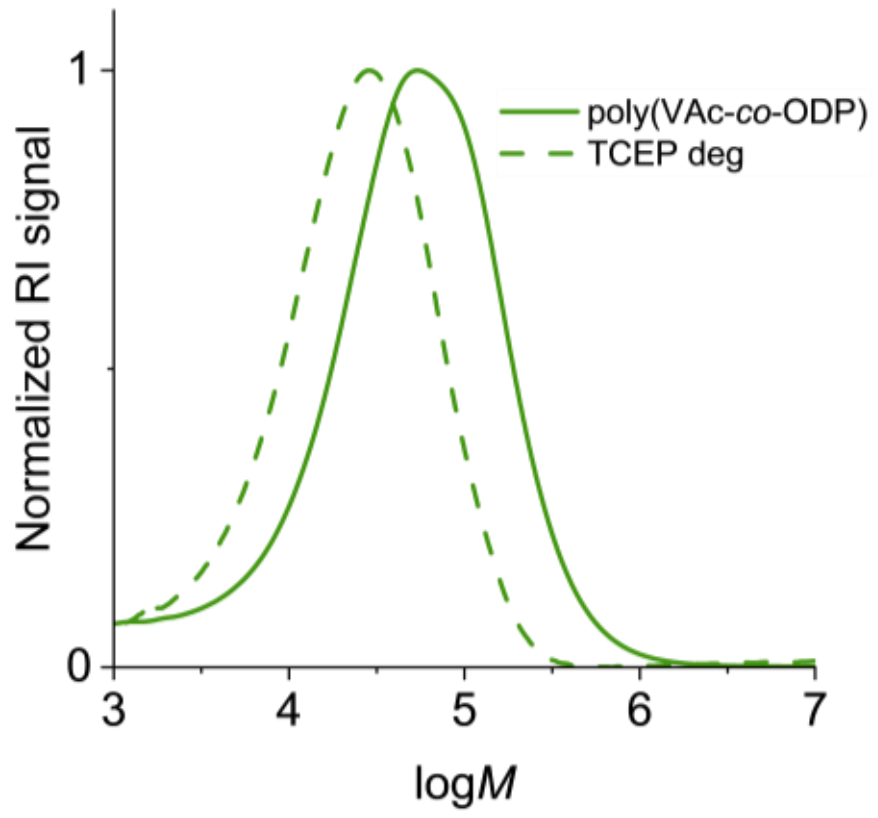

Figure S36. SEC traces recorded before and after degradation with TCEP of the copolymer of ODP with VAc, with initial comonomer feed $[VAc]_0:[ODP]_0$ = 95:5, prepared via FRP.

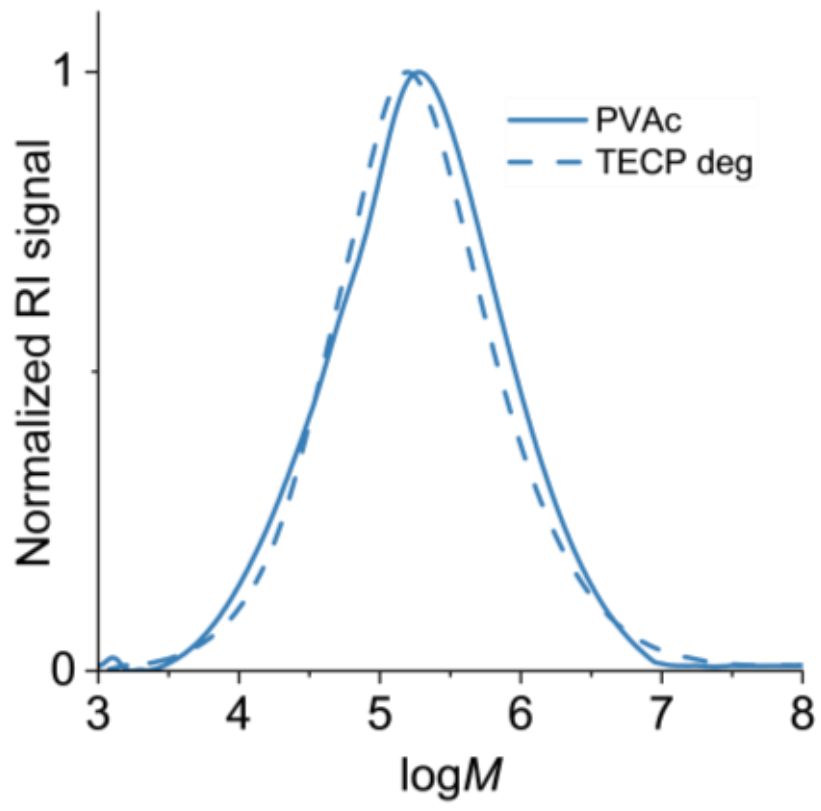

Figure S37. SEC traces recorded before and after degradation with TCEP of PVAc prepared via FRP.

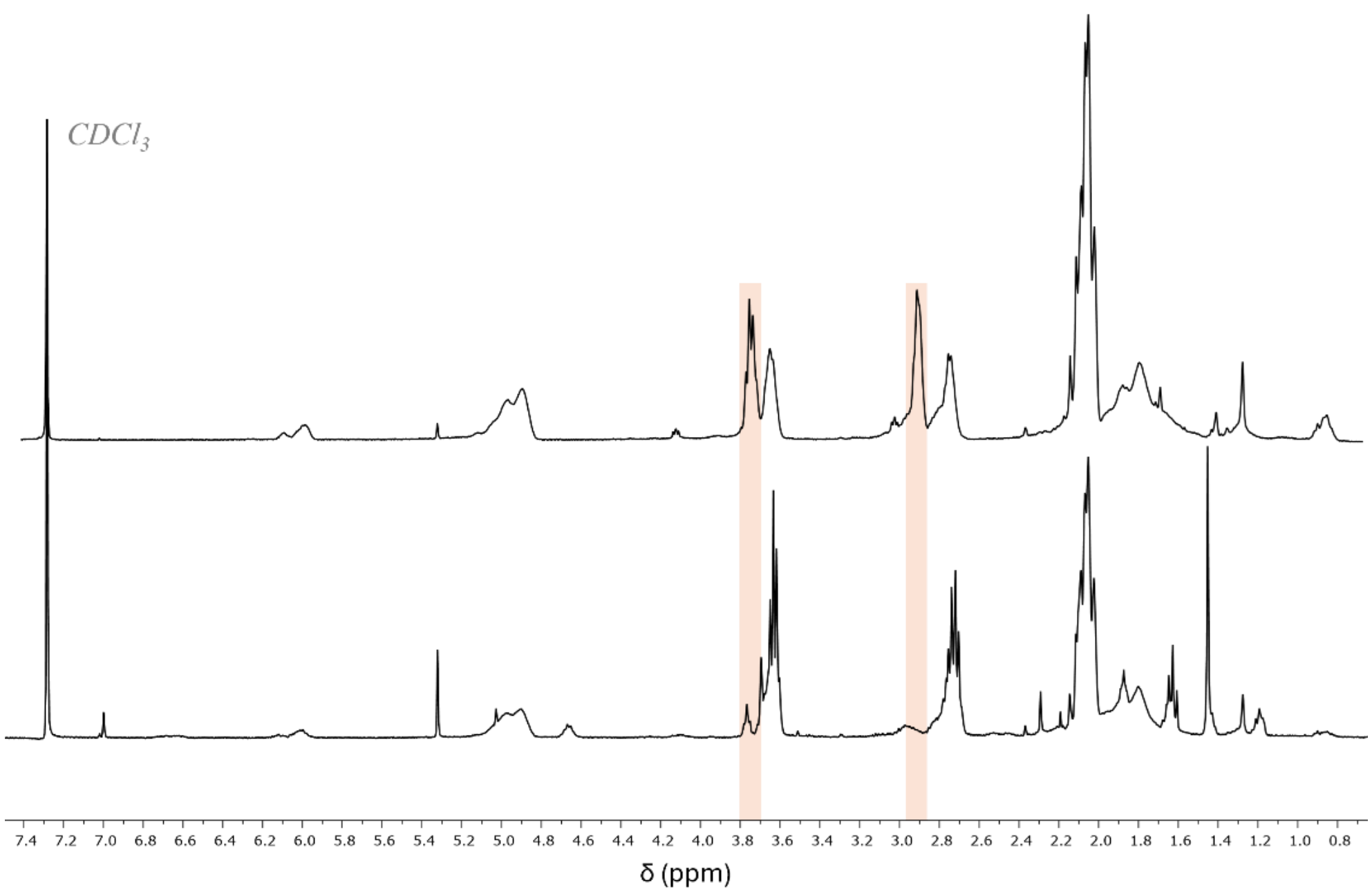

Figure S38. ^{1}H NMR spectra in $CDCl_3$ of P(VAc-*co*-ODC) before (top) and after (bottom) degradation with TCEP, which breaks the S-S bonds in the backbone.

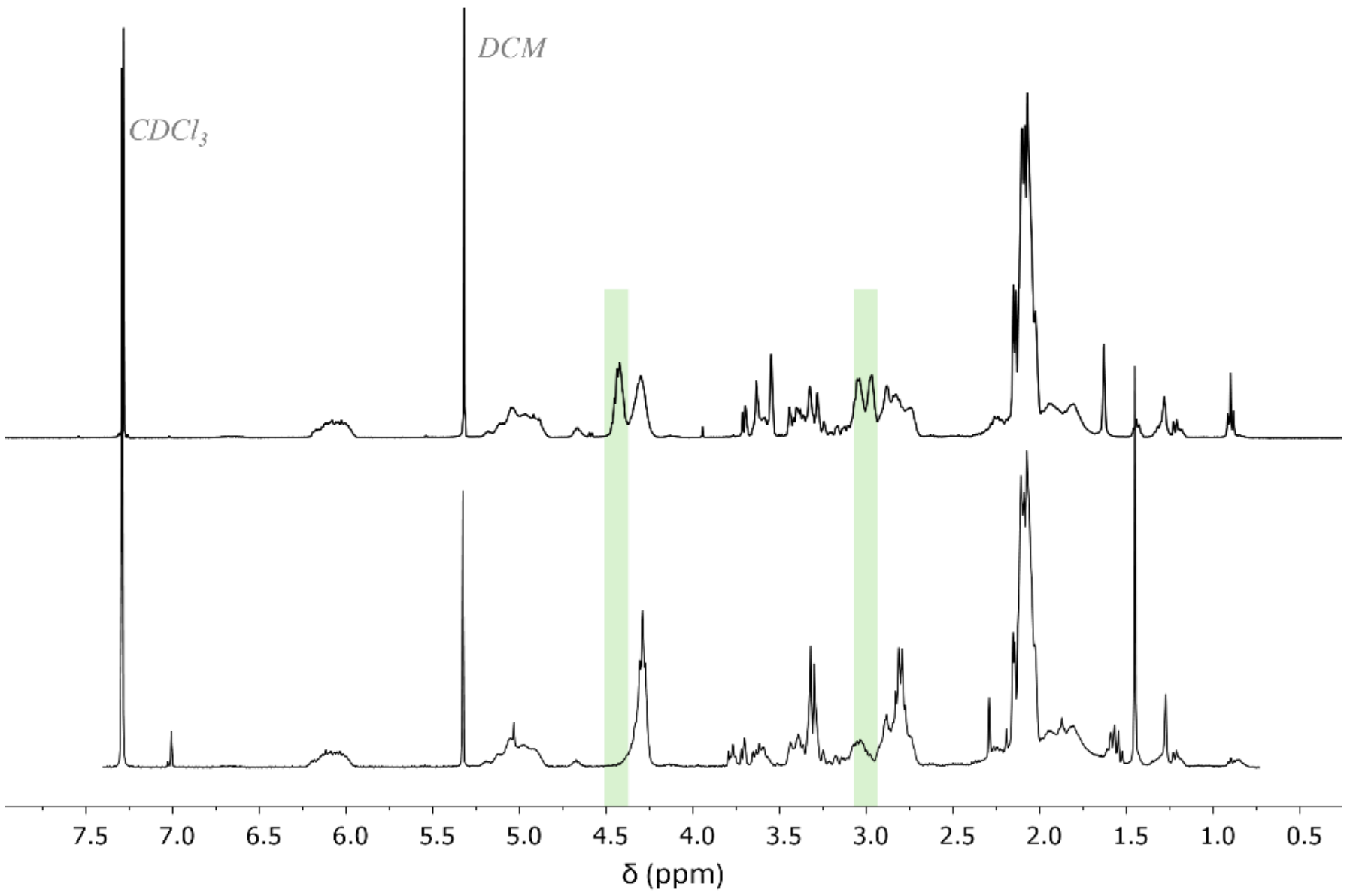

Figure S39. ^{1}H NMR spectra in $CDCl_3$ of P(VAc-*co*-ODP) before (top) and after (bottom) degradation with TCEP, which breaks the S-S bonds in the backbone.

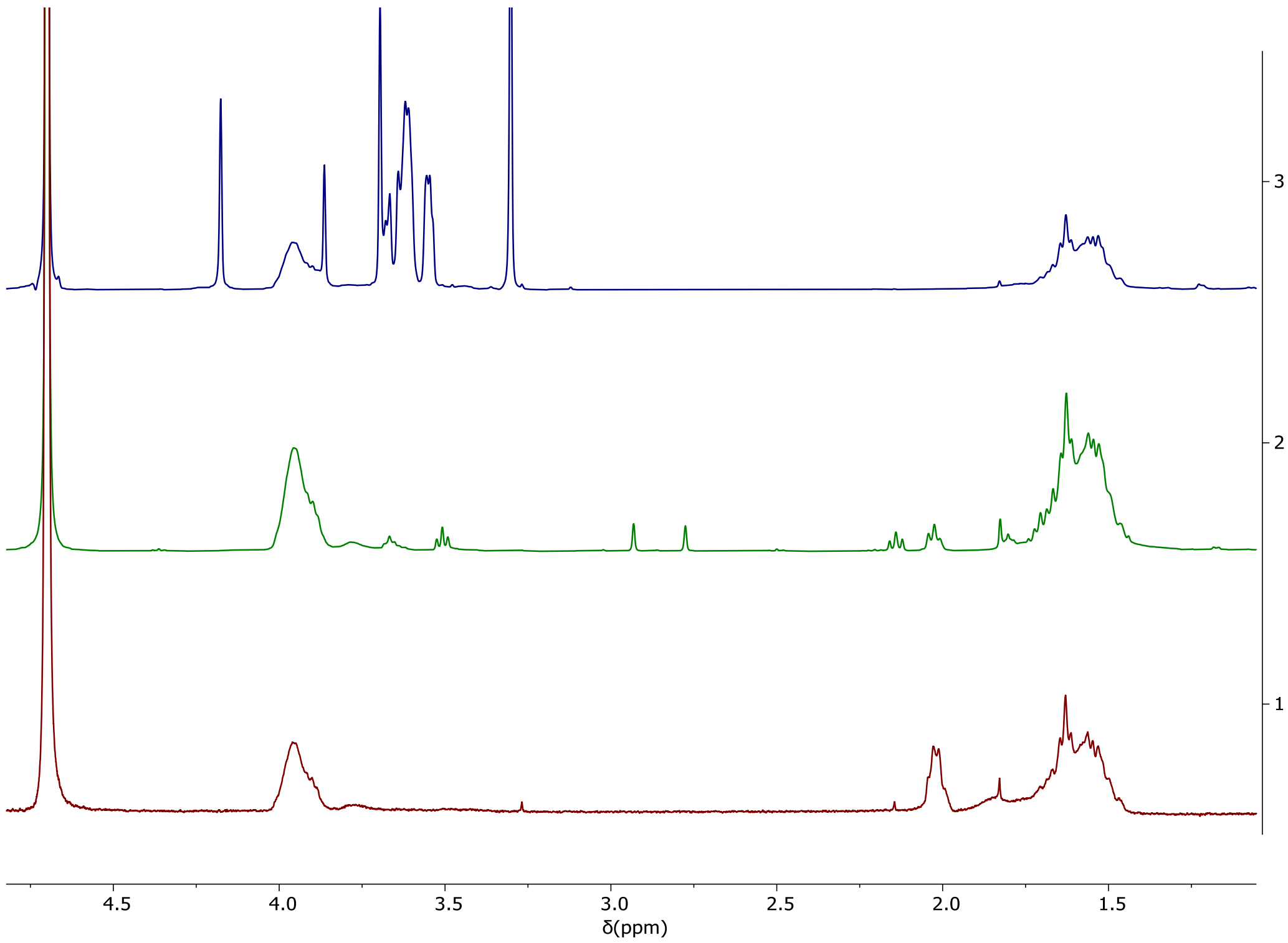

Figure S40. ^{1}H NMR spectra in D_2O of PDEGVE (blue trace) and PVAc (green trace) after degradation with 0.1 M KOH for 1.5 h. The spectrum of commercial poly(vinyl alcohol) (red trace) is reported as a reference.

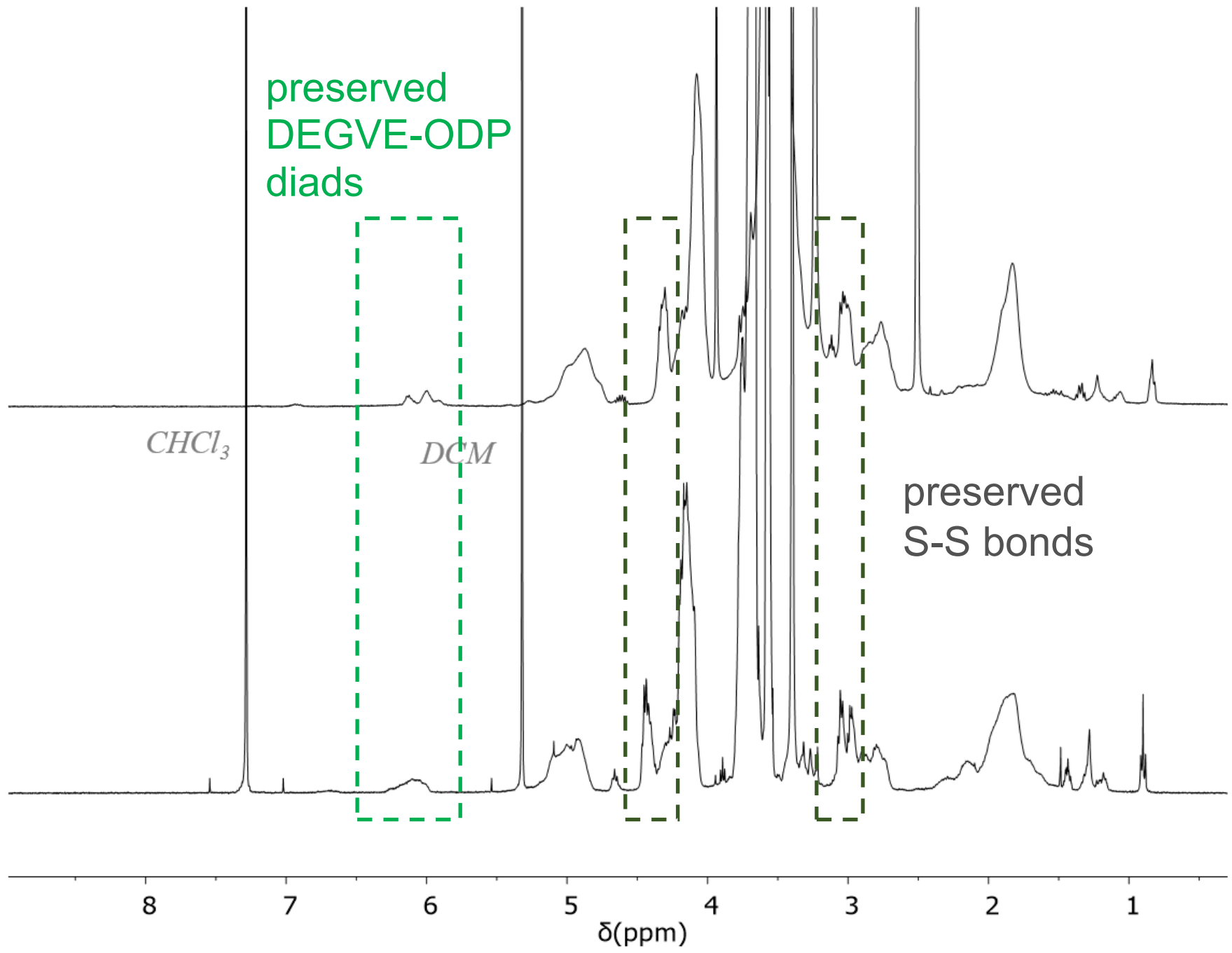

Figure S41. ^{1}H NMR spectra in $CDCl_3$ of P(DEGVE-*co*-ODP) after (top) and before (bottom) degradation in PBS, which breaks the ester bonds. The incorporation signal at δ ~6 ppm (dashed green line) remained visible, indicating that DEGVE-ODP diads were not altered during the degradation. Additionally, the degradation in PBS did not break the disulfide bonds (dashed dark green line).

Samples used for QCMD tests

Table S2. P(DEGVE-*co*-ODP) copolymers prepared via PI-RAFT/MADIX polymerization[a] and used for QCM-D tests.

Entry	$[M_{S-S}]_0$: $[M_{vin}]_0$	*t* (h)	Conv. M_{S-S} (%)[b]	Conv. M_{vin} (%)[b]	$[M_{S-S}]$: $[M_{vin}]$[c]	$M_{n,th}$[d] (kg mol^{-1})	M_n[e] (kg mol^{-1})	$Đ$[e]
1	5:95	30	70	28	14:86	9.1	8.9	1.56
2	15:85	28	81	23	38:62	8.9	6.5	1.47

[a] Copolymerizations were performed in bulk at T ~30 °C, with ($[M_{S-S}]+[M_{vin}]$):[CTA] = 150:1. Blu light irradiation: λ = 427 nm. [b] Determined by ^{1}H NMR. [c] Determined by ^{1}H NMR on the purified copolymers. [d] Calculated as $M_{n,th} = [(\text{Conv. } M_{S-S})\times DP_{T,S-S}\times MM_{S-S}] + [(\text{Conv. } M_{vin})\times DP_{T,vin}\times MM_{vin}] + MM_{CTA}$. [e] Measured by SEC using DMF (with 1 g L^{-1} LiBr) as eluent.
